# Modulation of metal-azolate frameworks for the tunable release of encapsulated glycosaminoglycans†

**DOI:** 10.1039/d0sc01204a

**Published:** 2020-07-14

**Authors:** Miriam de J. Velásquez-Hernández, Efwita Astria, Sarah Winkler, Weibin Liang, Helmar Wiltsche, Arpita Poddar, Ravi Shukla, Glenn Prestwich, John Paderi, Pablo Salcedo-Abraira, Heinz Amenitsch, Patricia Horcajada, Christian J. Doonan, Paolo Falcaro

**Affiliations:** Institute of Physical and Theoretical Chemistry, Graz University of Technology Stremayrgasse 9 Graz 8010 Austria paolo.falcaro@tugraz.at; School of Physical Sciences, Faculty of Sciences, University of Adelaide South Australia 5005 Australia christian.doonan@adelaide.edu.au; Institute of Analytical Chemistry and Food Chemistry, Graz University of Technology 8010 Graz Austria; School of Science, Nanobiotechnology Research Laboratory (NBRL), RMIT University 3001 Melbourne Australia; The University of Utah, College of Pharmacy Salt Lake City Utah 84112-5820 USA; Symic. Bio, Inc. 1400 Pine St., #640505 San Francisco CA 94164 USA; Advanced Porous Materials Unit (APMU), IMDEA Energy Avda. Ramón de la Sagra 3 E-28935 Móstoles Madrid Spain; Institute of Inorganic Chemistry, Graz University of Technology 8010 Graz Austria

## Abstract

Glycosaminoglycans (GAGs) are biomacromolecules necessary for the regulation of different biological functions. In medicine, GAGs are important commercial therapeutics widely used for the treatment of thrombosis, inflammation, osteoarthritis and wound healing. However, protocols for the encapsulation of GAGs in MOFs carriers are not yet available. Here, we successfully encapsulated GAG-based clinical drugs (heparin, hyaluronic acid, chondroitin sulfate, dermatan sulfate) and two new biotherapeutics in preclinical stage (GM-1111 and HepSYL proteoglycan) in three different pH-responsive metal-azolate frameworks (ZIF-8, ZIF-90, and MAF-7). The resultant GAG@MOF biocomposites present significant differences in terms of crystallinity, particle size, and spatial distribution of the cargo, which influences the drug-release kinetics upon applying an acidic stimulus. For a selected system, heparin@MOF, the released therapeutic retained its antithrombotic activity while the MOF shell effectively protects the drug from heparin lyase. By using different MOF shells, the present approach enables the preparation of GAG-based biocomposites with tunable properties such as encapsulation efficiency, protection and release.

## Abstract 2

Clinical and pre-clinical GAG-based biotherapeutics were encapsulated within three metal-azolate frameworks (ZIF-8, ZIF-90, and MAF-7). The resulting MOF biocomposites show different loading capacity, biopreservation properties and release profiles.

## Graphical abstract

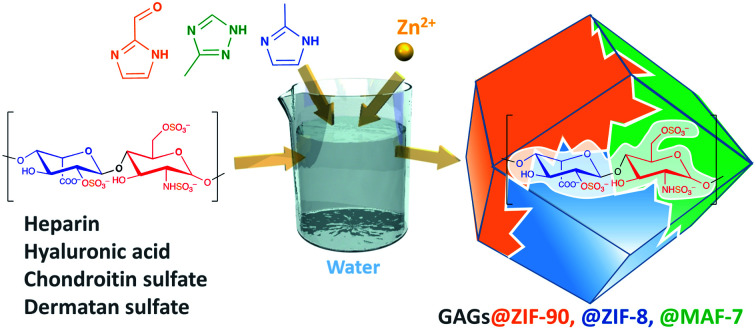

## Introduction

Metal–organic frameworks (MOFs) are a class of extended materials composed of metal nodes connected *via* multidentate organic linkers.^1,2^ The chemical mutability of these building blocks permits tailoring the properties of MOFs for applications ranging from gas storage to chemical sensing and catalysis.^3,4^ More recently, MOFs have been studied for drug delivery because of their high encapsulation efficiency, tunable release profile, high selectivity toward specific cells and tissues, and low cytotoxicity.^5^ MOFs can be loaded with different therapeutics, including biomacromolecules and assembly of thereof (proteins, DNA, viruses and cells).^6–8^ In these cases, the MOF coating acts as a protective carrier that can be dissolved under controlled conditions to release the bioentities.^7,9–12^ The immobilisation of large biomolecules within Zn-based zeolitic imidazolate frameworks (ZIFs),^13–16^ including ZIF-8 and related topologies, ZIF-90 and MAF-7,^17–20^ is commonly achieved *via* encapsulation.^6–8^ The widespread interest in ZIFs for the encapsulation of biomacromolecules is due to their compatibility with aqueous synthetic conditions,^6^ their hydrolytic stability,^21^ and their controlled release properties (*e.g. via* acidic pH or addition of chelating agents).^22^ These properties have been exploited for the design of stimulus-responsive drug delivery systems.^23–26^

Despite the versatility of the encapsulation protocol, we recently demonstrated that not all the biomacromolecules are prone to induce the spontaneous crystallisation of ZIFs.^27^ For example, negatively charged molecules trigger the growth of biocomposites, while their positively charged counterparts prevent their spontaneous formation.^27^ These results underscore the importance of electrostatic interactions between target biomolecules and Zn^2+^ ions, as an increased local concentrations of Zn^2+^ on the surface of the biomolecule triggers the self-assembly of the framework. We hypothesize that this approach can be applied to the design of ZIF-based drug carriers for highly-negative charged clinical biotherapeutics such as glycosaminoglycans,^28^ it is worth noting that, so far, carbohydrate@ZIF composites have been prepared only with CM-dextran (a model drug) and ZIF-8.^29^ Thus, the encapsulation of real carbohydrate-based therapeutics in azolate frameworks would progress MOF-based carriers to drug delivery applications. Glycosaminoglycans (GAGs) are unbranched high-molecular weight polysaccharides formed from disaccharide units that consist of an amino sugar (d-glucosamine or d-galactosamine), and uronic acid (d-glucuronic acid or l-iduronic acid).^28^ The multiple carboxylate and sulfate moieties attached to the carbohydrate backbone impart the negative charge to GAGs.^28^ The most common GAGs are heparin (HP), hyaluronic acid (HA), chondroitin sulfate (CS), and dermatan sulfate (DS).^28,30^ They naturally occur either covalently linked to proteins, forming proteoglycans, or free within the extracellular matrix.^28,30^ In living organisms, GAGs are involved in a variety of biological roles, including anti-coagulation, wound healing, lubrication of synovial joints, cell signalling, angiogenesis, and axonal growth.^28,30–32^ GAGs can be used as therapeutics to prevent the proliferation of bacteria (*e.g. Mycobacterium tuberculosis*), and viruses (*e.g. Herpes simplex*).^28,30–33^ Recently, the relevance of GAGs to vaccines, protein, and antibody modifications, and polyvalent glycan therapeutics has been highlighted by Paderi and co-workers.^34^ Furthermore, due to the important role of proteoglycans in tumour progression and metastasis, GAGs have been applied to the design of novel anticancer therapeutics.^28,30,32^

GAGs-based therapeutics are typically administered *via* the parenteral route as their bioavailability is compromised in the gastrointestinal tract.^28,35,36^ Dosing of GAGs, *via* the parenteral route requires careful monitoring, as an excess of the drug can lead to bleeding as result of their anticoagulant properties.^35,36^ This method of administration is not compatible with all disease treatments such as wound healing and anti-inflammatory applications that require efficient local administration.^37^ As a consequence, novel carriers with customisable delivery properties for the administration of GAGs are desirable.

This study presents a straightforward approach to circumvent those problems through the modulation of the drug release kinetics of the resultant biocomposites by tuning the physicochemical properties of the MOF shell. Three different Zn-based metal-azolate frameworks (ZIF-8, ZIF-90, and MAF-7), of markedly different hydro-phobicity/-philicity,^38,39^ were employed to encapsulate a selected set of GAGs-based therapeutics (HA, HP, CS, DS, GM-1111, and HepSYL, where the last two are synthetic drugs in preclinical development).^34,40,41^ The encapsulation efficiencies (EE%) and therapeutic release profiles of each biocomposite were assessed as these are crucial information for the development of drug delivery systems.^25^

As a case study, we focused on HP, a GAG with anticoagulant activity mediated by its affinity for binding to antithrombin III (AT) leading to the inhibition of serine proteases involved in the coagulation process.^42^ However, in this process, the therapeutic activity is strongly dependent on the preservation of specific pentasaccharide sequence of HP. Thus, subtle structural modifications on the pentasaccharide sequence might alter the anticoagulant activity of HP.^42^ In this context, the current delivery of HP is predominantly based on covalent surface immobilisation on carriers, an immobilisation method that compromises the activity of this GAG.^43^ Thus, new protocols for encapsulation and delivery of GAGs are highly desired.^42^ Here we examine the activity of HP released by HP@ZIF-8, HP@ZIF-90, and HP@MAF-7 demonstrating that MAF-7 fully preserve HP bioactivity.

For the first time we demonstrated that the encapsulation, protection and release of pharmacologically active carbohydrate-based therapeutics can be performed using azolate-based MOF particles.

## Results and discussion

**Scheme 1:**
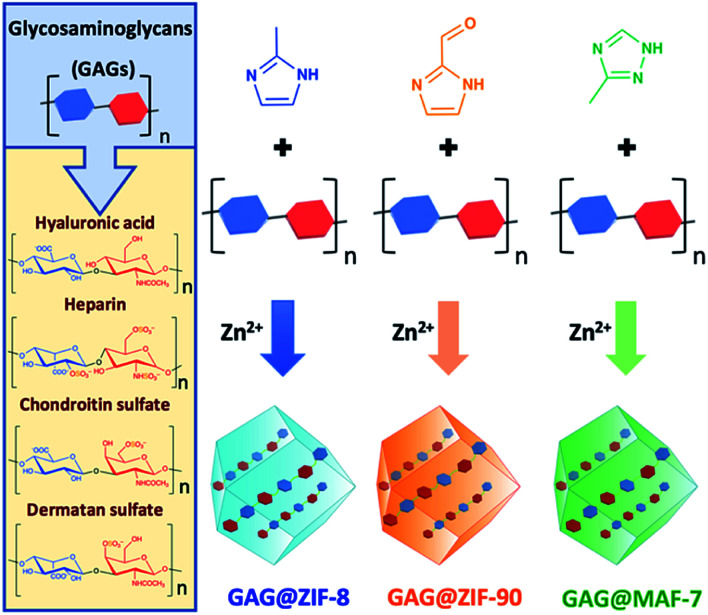
Schematic representation of one-pot synthesis of GAG@MOFs biocomposites based on three different metal-azolate frameworks.

ZIF-8-based biocomposites have been intensively studied for their drug release properties, however limited or no attention has been paid to ZIF-90 and MAF-7 as drug carriers.^25,44,45^ Although, ZIF-8, ZIF-90 and MAF-7 are isoreticular, they are composed of different organic linkers and possess distinct chemical properties.^17,38,39^ For example, ZIF-8 (2-methylimidazole; HmIM) is more hydrophobic than ZIF-90 (2-imidazole carboxaldehyde; HICA) and MAF-7 (3-methyl-1,2,4-triazole; Hmtz).^38,39^ We posit that the different properties of these ZIFs could influence their performance as drug delivery carriers. To verify this hypothesis, we encapsulated six different GAGs-based therapeutics (HA, HP, CS, DS, GM-1111, and HepSYL) within ZIF-8, ZIF-90, and MAF-7, respectively (Scheme 1) and examined the performance characteristics of the GAG@ZIF biocomposites as carriers for pH-responsive delivery. For each system, we determined the encapsulation efficiency (EE%) and the drug release profiles.^46^ However, we first focused our attention on finding the synthetic conditions to the loading capacity and release properties of the biocomposites derived from the three different MOF systems (ZIF-8, ZIF-90, and MAF-7).

Given that there are no previous studies describing the encapsulation of carbohydrates in ZIF-90 and MAF-7, we used carboxymethyl-dextran tagged with fluorescein isothiocyanate (FITC–CMD), as a model therapeutic to determine if carbohydrate-based biocomposites of these ZIFs could be obtained. FITC–CMD was selected as it is an inexpensive carbohydrate that closely mimics GAGs, and the fluorescein tag permits quantification of the amount of CM-dextran encapsulated (Fig. S1–S3, ESI†).^22^

The synthesis of FITC–CMD@ZIF-8, FITC–CMD@ZIF-90 and FITC–CMD@MAF-7 was performed by varying the concentration of the biomolecule ([FITC–CMD] = 0 (**1**), 0.18 (**2**), 0.36 (**3**), 0.72 (**4**), 1.44 (**5**) mg mL^−1^) and the metal to ligand ratio (Zn^2+^ : L = 1 : 4 (**A**), 1 : 3.47 (**B**) and 1 : 2.52 (**C**)) (Tables S1 and S2†). The data shows that for FITC–CMD@ZIF-8 biocomposites, the optimal encapsulation efficiencies (>90%) were reached using 0.36 and 0.72 mg mL^−1^ of FITC–CMD and metal to ligand ratios of 1 : 4 (**A**) and 1 : 3.47 (**B**) (Fig. S1, ESI†). In the case of FITC–CMD@ZIF-90 biocomposites the higher EE% values (>90%) were observed for samples obtained from the lowest concentration of FITC–CMD (0.18 mg mL^−1^), for both 1 : 3.47 (**B**) and 1 : 2.52 (**C**) Zn^2+^ : HICA ratios (Fig. S2, ESI†). Conversely, FITC–CMD@MAF-7 biocomposites present exceptional polysaccharide payloads regardless of the initial concentration of FITC–CMD, when using 1 : 3.47 (**B**) Zn^2+^ : Hmtz ratio (Fig. S3, ESI†). However, It should be pointed out that, unlike previous reports describing the synthesis of protein@MAF-7 biocomposites,^19,38^ in this work FITC–CMD@MAF-7 is synthesised in absence of ammonia, a deprotonating agent with low biocompatibility.^47^ Furthermore, we note that the encapsulation efficiency of FITC–CMD increases concomitantly with a reduction in the amount of NH_3_·H_2_O.

The drug release kinetics of the FITC–CMD@ZIF-8, FITC–CMD@ZIF-90 and FITC–CMD@MAF-7 biocomposites were obtained upon applying an external acidic stimulus (Fig. S4–S6, ESI†). The release profiles obtained from FITC–CMD@MAF-7 biocomposites show that the higher the concentration of the FITC–CMD the faster the delivery of the cargo (Fig. S7†). Similar behaviour was observed for the samples obtained from FITC–CMD@ZIF-90 when using 1 : 3.47 metal-to-ligand ratio (**90DXBn**; where *n* = **2**–**5**). However, for FITC–CMD@ZIF-90 biocomposites obtained from Zn^2+^ : HICA = 1 : 4 and Zn^2+^ : HICA = 1 : 2.52 ratios (**90DXAn** and **90DXCn**, respectively; where *n* = **2**–**5**), as well as for the FITC–CMD@ZIF-8 biocomposites, a clear trend is not evident (Fig. S7†). For FITC–CMD@MAF-7 and FITC–CMD@ZIF-90 biocomposites, the release rate increases as the Zn^2+^ : L ratio decreases (Fig. S4–S7†). For FITC–CMD@ZIF-8 biocomposites, this trend was observed only for the samples obtained with FITC–CMD > 0.36 mg mL^−1^; however, the FITC–CMD@ZIF-8 samples obtained with FITC–CMD = 0.18 mg mL^−1^ show the slowest release rate with Zn^2+^ : HmIM = 1 : 3.47 (Fig. S4–S7†).

Additionally, our stability tests in water (pH = 7.0) demonstrate that for FITC–CMD@MOF biocomposites obtained from 0.36 mg mL^−1^ of FITC–CMD keeping the Zn^2+^ : L = 1 : 3.47 (**8DXB3**, **90DXB3** and **7DXB3**) do not release FITC–CMD after being stored in water (pH = 7.0) for 24 h (Fig. S24, ESI†). However, for its analogues obtained from 0.72 mg mL^−1^ and 1.44 mg mL^−1^ of FITC–CMD we measured leaching of the model drug during the incubation of the samples in DI water for 24 h at room temperature.

Thus, the maximum concentration of FITC–CMD that afforded controlled stimulus-response drug release was 0.36 mg mL^−1^. The stability of the samples **8DXB3**, **90DXB3** and **7DXB3** was further confirmed by inductively coupled plasma-optical emission spectrometry (ICP-OES) through the determination of Zn^2+^ released upon the incubation of the samples in DI water for 24 h (Fig. S24d, ESI†).

In summary, employing FITC–CMD, as a model drug, we found that a metal to ligand ratio Zn^2+^ : L = 1 : 3.47 and a carbohydrate concentration of 0.36 mg mL^−1^ yielded acceptable EE% and facilitates the release of the model therapeutic on demand. Accordingly, these synthetic conditions (Zn^2+^ : L = 1 : 3.47, [GAG] = 0.36 mg mL^−1^) were employed for the encapsulation of selected GAG-based therapeutics (*i.e.* HA, HP, CS, DS, see Scheme 1).

The biocomposites derived from ZIF-8 and ZIF-90 were isolated as crystalline precipitates (HA@ZIF-*n*, HP@ZIF-*n*, CS@ZIF-*n*, and DS@ZIF-*n*, where *n* = 8 and 90; respectively). However, the MAF-7-based biocomposites formed either viscous solutions with a gel-like consistency (HP@MAF-7, CS@MAF-7, and DS@MAF-7) or non-flowing gels (HA@MAF-7). The formation of metal–organic gels has been previously explained as a result of the rapid formation of MOF nanoparticles, which aggregate through weak van der Waals interactions, H-bonding or π–π stacking.^48–50^

**Fig. 1:**
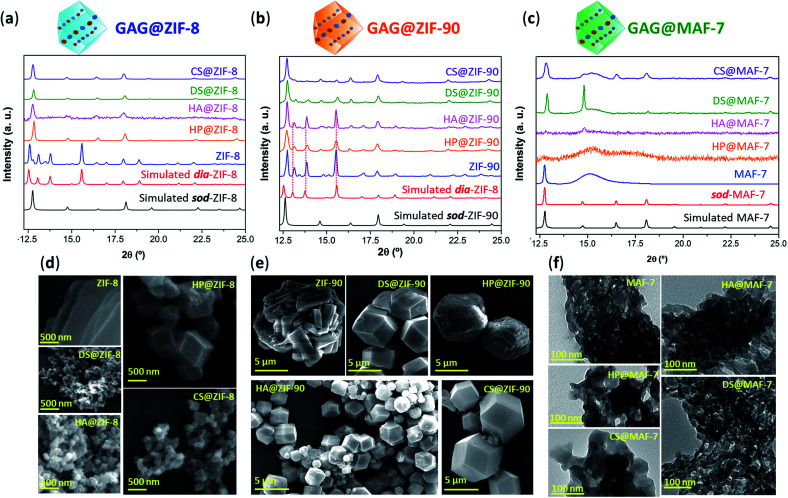
(a) Comparison of the diffraction patterns of ZIF-8 and GAG@ZIF-8 biocomposites (GAGs = heparin (HP), hyaluronic acid (HA), chondroitin sulfate (CS), and dermatan sulfate (DS)). (b) Diffraction patterns obtained from ZIF-90 and GAG@ZIF-90 biocomposites. The dashed lines represent the diffraction peaks associated to the formation of **dia** phase. (c) Diffraction patterns of **sod**-MAF-7 obtained in presence of NH_3_·H_2_O (10%), as well as the air-dried xerogel obtained from the synthesis of MAF-7 and GAG@MAF-7 biocomposites without ammonia. (d) SEM images of pure ZIF-8 and its corresponding GAG@ZIF-8 biocomposites. (e) SEM images of ZIF-90 and GAG@ZIF-90 biocomposites. (f) TEM images of MAF-7 and GAG@MAF-7 biocomposites obtained without the addition of deprotonating agents (*e.g.* NH_3_·H_2_O).

After the 24 h of reaction, GAG@MOF samples were washed with water and ethanol (see ESI† for details) and the air-dried solids were analysed by powder X-ray diffraction (PXRD) (Fig. 1). The diffraction patterns show that the control sample (ZIF-8 without biotherapeutic) possess predominantly a diamondoid topology (**dia**), while the GAG@ZIF-8 biocomposites exhibit a sodalite (**sod**) topology (Fig. 1a). These data indicate that the presence of GAGs enhance the formation rate of the ZIF yielding the less thermodynamically stable **sod** topology.^51^ The PXRD pattern of ZIF-90, prepared in the absence of GAGs, is consistent with the **dia** polymorph. However, in presence of the biomolecule the resultant GAG@ZIF-90 biocomposites show a mixture of kinetic **sod**-ZIF-90 and the thermodynamic polymorph **dia**-ZIF-90 (Fig. 1b). Finally, the diffraction pattern of pure MAF-7 synthesised in presence of NH_3_·H_2_O (10%) shows the formation of a crystalline phase with **sod** topology, which is consistent with previous reports.^19,38^ Conversely, the PXRD pattern of MAF-7 prepared in absence of ammonia shows diminished crystallinity (Fig. 1c). The diffraction patterns obtained from the GAG@MAF-7 biocomposites indicate that HA@MAF-7 and HP@MAF-7 are predominantly amorphous, whereas DS@MAF-7 shows a mixture of crystalline and amorphous phase. The sample CS@MAF-7 gives rise to a PXRD pattern with broad diffraction peaks that can be attributed to the presence of nanoparticles with domain sizes between (3.3 ± 2 to 92.7 ± 5 nm) as determined by the Scherrer equation (Fig. 1c). The crystal size and the morphology of the control samples of MOFs and their corresponding GAG@MOF biocomposites were assessed by scanning electron microscopy (SEM) (Fig. 1). The micrographs obtained from the control sample of ZIF-8 show the formation of plate-like crystals, which is the typical morphology observed in **dia**-ZIF-8 topology (Fig. S8†).^18,20,51^ For GAG@ZIF-8 (Fig. 1d), the characteristic rhombic dodecahedron morphology of **sod**-ZIF-8 topology is present.^18,20,51^ HA@ZIF-8, CS@ZIF-8, and DS@ZIF-8 have particle sizes below 500 nm, while HP@ZIF-8 shows wider size distribution up to 1 μm (Fig. 1d, S8 and S9†).

SEM images obtained from pure ZIF-90 shows spherical clusters of prismatic crystals (Fig. 1e). For all the other samples prepared in presence of GAGs (GAG@ZIF-90) a rhombic dodecahedron morphology is observed (Fig. 1e).^18^ The crystalline powder obtained from HP@ZIF-90, CS@ZIF-90, and DS@ZIF-90 possesses particle sizes ranging from *ca.* 5 μm to *ca.* 7 μm; whereas HA@ZIF-90 presents a wider particle size distribution ranging from *ca.* 500 nm to *ca.* 4 μm (Fig. S10 and S11, ESI†). Furthermore, mesopores are observed on the surface of some of the GAG@ZIF-90 crystals. Although this is more evident for HP@ZIF-90, this can be seen for CS@ZIF-90, and DS@ZIF-90 (Fig. 1e and S11†). Similar textural features have been previously found in other MOFs, prepared in the presence of long-chain carboxylic acids.^52,53^ Due to the small particle sizes of the MAF-7 materials obtained without NH_3_·H_2_O (10%), they were studied using transmission electron microscopy (TEM) (Fig. 1f). The images show that the solid materials obtained for pure MAF-7 and GAGs@MAF-7 consist of aggregated nanoparticles with an average size below 100 nm (Fig. S12 and S13†).

To ascertain the encapsulation of GAGs within the ZIF matrices, the samples were washed with water (2 mL, 3×) and ethanol (2 mL, 3×) to ensure the complete removal of GAGs loosely attached to the particle surface.^22^ Then the collected solids were analysed by Fourier transform infrared spectroscopy (FTIR) (Fig. S14–S18†). IR spectra obtained from GAGs@ZIFs biocomposites show the vibration bands typically attributed to the ZIF framework including the Zn–N stretching mode (421 cm^−1^) and characteristic vibrational modes of the azolate ligands (1584 cm^−1^ (*ν*_C=N_), 1500–1350 cm^−1^ (*ν*_ring_) and 800–650 cm^−1^ (*δ*_ring_)) (Fig. S14†). For each GAG used, additional bands originating from the specific pendant groups were observed.^54^ For example, the vibrational bands attributed to the carboxylic groups are found around 1610–1620 cm^−1^*ν*_as_(COO^−^) and 1410–1420 cm^−1^*ν*_s_(COO^−^). Furthermore, all of the spectra also display a broad band around 2850–3600 cm^−1^, that results from the stretching modes of the OH group, as well as the band attributed to C–O stretching vibration in 1020–1040 cm^−1^.^54^

Finally, those biocomposites obtained from sulfated biomacromolecules (HP, CS, and DS) present additional weak vibrational bands at 1220–1240 cm^−1^*ν*_as_(S=O), and 1000–822 cm^−1^ (OSO_3_^−^) (Fig. S16–S18†).^54^

**Fig. 2:**
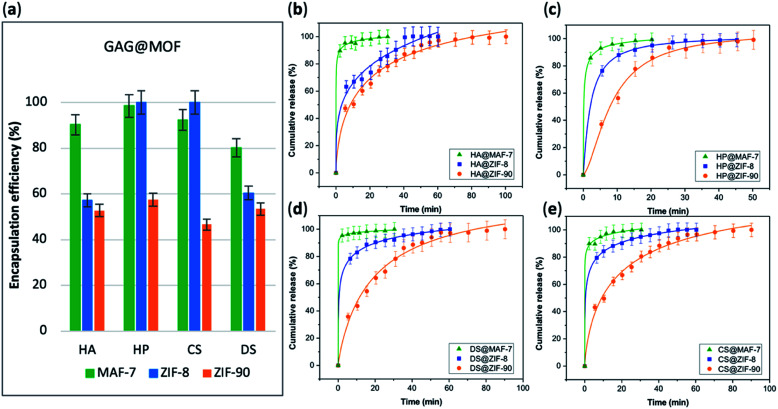
(a) Encapsulation efficiency (%) of the GAGs-based biocomposites based on three different MOFs (ZIF-8, ZIF-90 and MAF-7). Comparative release profiles of the biocomposites: (b) HA@MOFs, (c) HP@MOFs, (d) DS@MOFs, and (e) CS@MOFs, upon applying an acidic stimulus (pH = 6).

The EE% of each GAG@MOF biocomposite was assessed using UV-vis spectroscopy using the carbazole assay; which is a direct method to quantify glycosaminoglycans by colorimetry (*λ*_max_ = 520 nm) (see ESI† for details).^55,56^ The GAG@MOF samples, were soaked, separately, in citrate buffer (2 mL, 80 mM, pH = 6) to dissolve the MOFs. Once a clear solution was obtained, a Sephadex column was used to separate the GAGs from the MOF precursors. For these clinical biotherapeutics, the MAF-7-based biocomposites present the highest EE% reaching values above 80% (Fig. 2a) and the GAG@ZIF-90 biocomposites display the lowest EE% (*ca.* 50%) (Fig. 2a). In the case of ZIF-8, the biocomposites obtained from HP and CS present exceptional EE% (*ca.* 100%); however, those derived from HA and DS show an EE% of *ca.* 60% (Fig. 2a).

The amount of the commercial GAGs (HP, CS, DS, HA) encapsulated in MOFs was confirmed by thermogravimetric analysis (TGA) (Fig. S28, ESI†) and high loading capacity of HP were calculated (*e.g.* 19 wt% HP for HP@MAF-7).

The drug-release profile studies were determined by quantifying the amount of GAG delivered in citrate buffer (80 mM, pH = 6) as a function of time (see ESI†). The citrate buffer was employed with the aim of emulating the interstitial tissue pH found in inflammatory diseases and in cancer cells.^57,58^ All the release profiles present an initial rapid release of the biotherapeutic, followed by a slower sustained delivery. Nevertheless, each MOF-system shows unique release behaviour (Fig. 2b–e). For instance, the release profiles of GAG@MAF-7 biocomposites exhibit a large initial burst release, where *ca.* 50% of the cargo was liberated within the first minute, reaching the complete delivery within 30 min (Fig. S19†). The observed burst effect for MAF-7-based biocomposites, irrespective of the GAG used, can be explained by the rapid degradation of the small nanoparticles. In the case of GAG@ZIF-8 biocomposites, the initial release rate in the burst stage varies: CS@ZIF-8 and DS@ZIF-8 present the fastest initial drug release (*ca.* 50% within the first minute), reaching the complete delivery of the cargo after 1 h (Fig. 2d and e).

HP@ZIF-8 and HA@ZIF-8 show a 50% release of the cargo within 5 min, and 100% release after 40 min (Fig. 2b and c). GAG@ZIF-90 composites display a sustained longer-term release profile. The initial burst stage is observed in 10 to 15 min, and *ca.* 50% of the loaded drug was released, followed by a gradual delivery of the cargo where the complete release ranges from 50 min (HP@ZIF-90) to 1.5 h (HA@ZIF-90, CS@ZIF-90 and DS@ZIF-90) (Fig. 2 and S19†).

In summary, by using different azolate-based MOFs we proved that we can design systems for the customised release of carbohydrate-based therapeutics from fast delivery, useful in case of infections,^59^ to longer delivery desired in case of anticoagulant administration.^60^ For example, for heparin, the poor dosage control *via* intravenous administration could lead to either fast clearance from the body (under-dosage) or spontaneous haemorrhages (over-dosage).^61,62^ An initial rapid release of HP followed by a more sustained delivery is most suitable for the treatment of urgent clinical situations, such as vascular surgery, frostbite, dialysis, *etc.*^37,43,63^ Thus, the development of HP delivery systems with fast responsive rate have attracted significant attention.^64–66^ The here prepared HP@MOF composites show release profiles that are relevant for urgent medical treatments.^37,43,63^

**Fig. 3:**
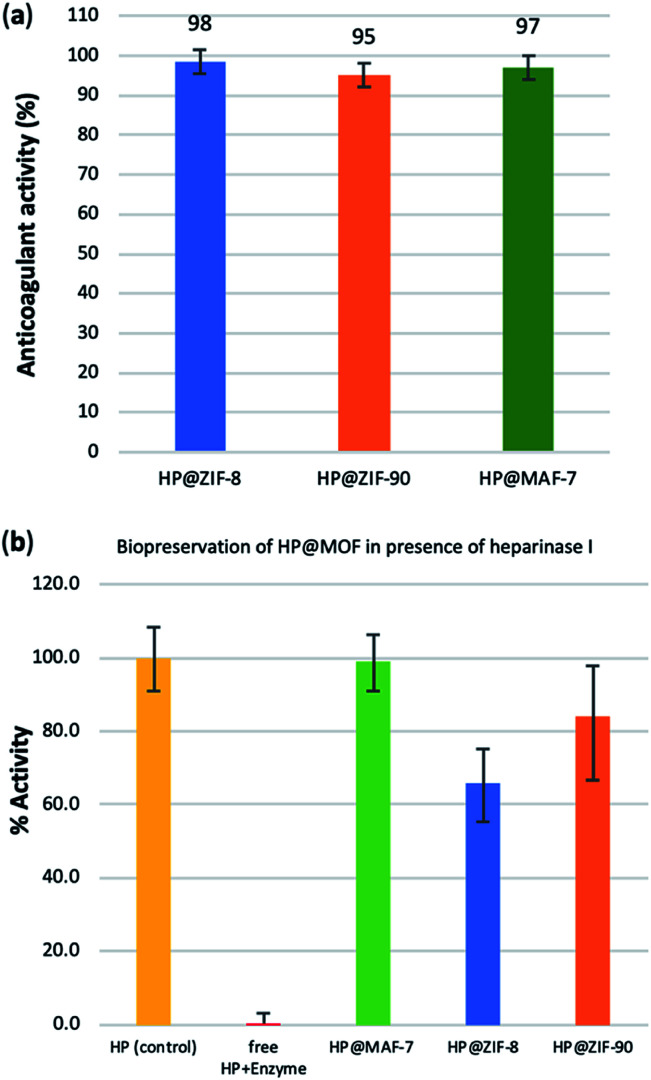
(a) Determination of the remaining anticoagulant activity of the HP released from the HP@MOFs biocomposites. (b) Comparison of the anticoagulant activity of unprotected HP and HP encapsulated within the MOF particles after being exposed to heparinase I.

To test possible alteration in the biotherapeutic properties of HP due the encapsulation and recovery processes, we used a chromogenic anti-IIa assay to evaluate the anticoagulant activity of heparin before and after being encapsulated within the three different MOFs (see ESI† for details). The collected data reveals that the HP released from ZIF-8 retains ≈98% of its initial activity, whereas the HP released from ZIF-90 and MAF-7 retains ≈95% and ≈97% activity, respectively (Fig. 3a and S26, ESI†). To verify the successful encapsulation of HP, we exposed the biocomposites to heparinase I, which is a heparin lyase over expressed in infected human organs and tissues.^67^ Thus, herein, HP@MOFs biocomposites and the free HP were exposed to heparinase I for 1 h at 30 °C. Subsequently, the encapsulated HP was recovered from the HP@MOF biocomposites and the anticoagulant activity was determined using anti-IIa chromogenic assay and compared with the activity of unprotected heparin exposed to the enzyme and pure HP as a control (Fig. S26, ESI†). The results showed that the unprotected heparin loses completely its anticoagulant activity. In contrast, the HP released from the HP@ZIF-8 and HP@ZIF-90 partially retains the antithrombotic activity (≈67%, ≈84%; respectively), whereas the activity HP released from MAF-7 is fully preserved (≈99%) (Fig. 3b). These results demonstrate that HP is predominantly located within the MOF shells that protect HP from lyases.

**Fig. 4:**
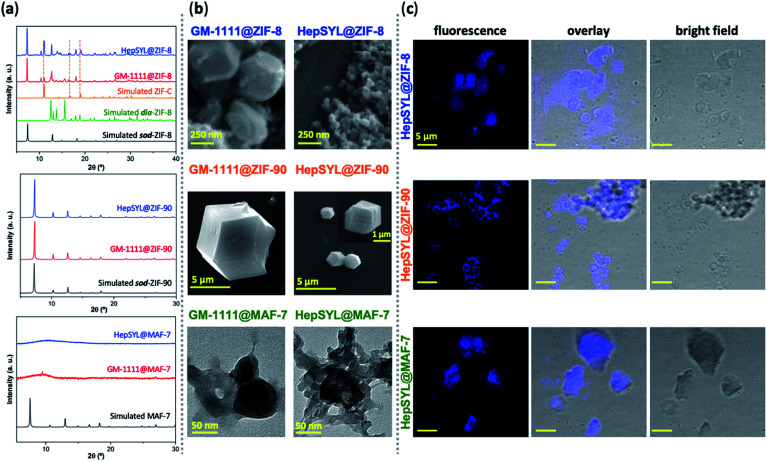
(a) PXRD patterns of HepSYL@MOFs and GM-1111@MOFs based on three different MOF systems (ZIF-8, ZIF-90 and MAF-7). The dashed lines represent the diffraction peaks associated to the formation of a **ZIF-C** phase. (b) SEM images of HepSYL@ZIF-8, GM-1111@ZIF-8, HepSYL@ZIF-90, and GM-1111@ZIF-90 biocomposites; TEM micrographs of HepSYL@MAF-7 and GM-1111@MAF-7. (c) Confocal laser scanning micrographs showing the fluorescence, bright field, and overlay images of HepSYL@ZIF-8, HepSYL@ZIF-90, and HepSYL@MAF-7 (scale bar: 5 μm).

To this point we have established that carbohydrate-based drugs can be encapsulated with high efficiency and their release can be controlled by the judicious selection of the MOF matrix (ZIF-8, ZIF-90 and MAF-7), we expanded our study to the assessment of ZIFs for the delivery of carbohydrate-based drugs in late-stage clinical trials. Thus, we employed two preclinical stage biotherapeutics: GM-1111 and HepSYL.^34,40,41^ GM-1111 is an anti-inflammatory agent engineered to treat chronic rhinosinusitis: it inhibits multiple inflammatory mediators and requires topical intranasal administration route.^68^ HepSYL is a new synthetic proteoglycan designed for oncotherapy applications. As such, a parenteral administration route is needed. GM-1111 and HepSYL were encapsulated within ZIF-8, ZIF-90 and MAF-7 following the synthetic protocol used for the GAGs based therapeutics (see ESI†). After washing and drying, the powders were examined with PXRD. The diffraction patterns indicate that GM-1111@ZIF-8 and HepSYL@ZIF-8 are a mixture of different crystalline phases, **sod**, **dia**, and **ZIF-C**,^20,69^ with **sod** as the predominant phase (Fig. 4a). By contrast, the diffraction pattern of GM-1111@ZIF-90 and HepSYL@ZIF-90 show that the samples are pure **sod** phase (Fig. 4a). GM-1111@MAF-7 and HepSYL@MAF-7 yield amorphous materials (Fig. 4a).

SEM analysis reveals that the crystalline particles of GM-1111@ZIF-8 and HepSYL@ZIF-8 are of rhombic dodecahedron morphology (Fig. 4b). For GM-1111@ZIF-8 we observed inhomogeneous particles with average size of *ca.* 500 nm; while for HepSYL@ZIF-8 the particles are homogeneous with size is below 200 nm. Likewise, the particle morphology observed in GM-1111@ZIF-90 and HepSYL@ZIF-90 samples corresponds to rhombic dodecahedron, with particle sizes of *ca.* 8 μm and *ca.* 2 μm, respectively (Fig. 4b). Due to the small particle size, GM-1111@MAF-7 and HepSYL@MAF-7 samples were analysed by TEM (Fig. 4b). The images reveal the formation of aggregates comprised of nanoparticles with an average size below 50 nm. Finally, confocal laser microscopy (CLSM) was employed to ascertain the location of HepSYL within the ZIF particles (Fig. 4c, S20, and S21†). The CLSM images show that the proteoglycan is homogeneously distributed within ZIF-8 and MAF-7 (Fig. 4c, and S20†). However, in the case of HepSYL@ZIF-90, the proteoglycan is predominantly localised towards the surface region of crystalline particles (Fig. 4c and S20†).

**Fig. 5:**
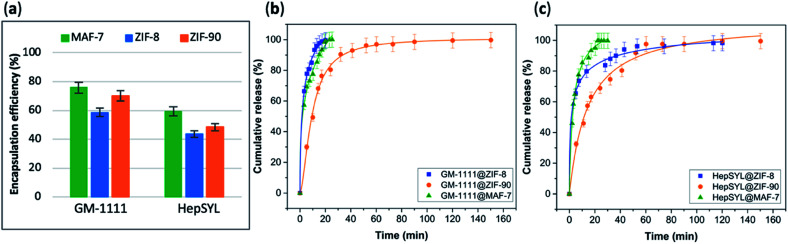
(a) Encapsulation efficiency (%) of HepSYL@MOFs and GM-1111@MOFs biocomposites obtained using the three different MOF systems (ZIF-8, ZIF-90 and MAF-7). Comparative release profiles of the biocomposites: (b) GM-1111@MOFs, and (c) HepSYL@MOFs, upon applying an acidic stimulus (pH = 6).

The EE% and the drug-release kinetics of GM-1111@MOF and HepSYL@MOF were assessed using UV-vis spectroscopy (Fig. 5a). Following the protocol previously described for GAG-based biocomposites, the amount of GM-1111 encapsulated within the MOF shell was determined using the carbazole assay (*λ*_max_ = 520 nm).^55,56^

In the case of HepSYL@ZIFs biocomposites, the EE% was determined by monitoring the absorbance of the colorant used to label the protein (CF633, *λ* = 633 nm) (Fig. S22†).

The data collected reveals that the best performance, in terms of EE%, was found when using MAF-7, followed by ZIF-90 and then ZIF-8 (Fig. 5a). The EE% is also influenced by the biomacromolecule. GM-1111, that is more structurally similar to GAGs, shows a higher EE% than HepSYL, which contains positively charged peptides.

The drug release profiles of GM-1111@ZIFs reveal that GM-1111@MAF-7 and GM-1111@ZIF-8 present a rapid burst release upon applying an external acidic stimulus (pH = 6), and complete release was achieved within the first 20 min (Fig. 5b).

The release profile of GM-1111@ZIF-90 presents a long-term controlled drug delivery, with complete release observed after 2.5 h (Fig. 5b). Finally, the HepSYL@ZIF release profiles reveal that the fastest drug delivery is determined for HepSYL@MAF-7, while HepSYL@ZIF-8 and HepSYL@ZIF-90 exhibit a longer-term drug release (Fig. 5c). The complete release of the anticarcinogenic therapeutic was observed after 2 h and 2.5 h, respectively (Fig. 5c).

## Conclusions

In summary, three chemically different metal-azolate based frameworks (ZIF-8, ZIF-90, and MAF-7), were used to design pH-responsive carriers for the encapsulation and release of GAG-based biotherapeutics including heparin (HP), chondroitin sulfate (CS), dermatan sulfate (DS), and hyaluronic acid (HA). Based on the selection of the ZIF matrices, the encapsulation efficiency varied from 50 to 100%, and the release of the clinical therapeutics could be precisely tailored. For instance, where a fast release is desired (*e.g.* in the treatment of infection-related diseases as the case of osteomyelitis,^70^ in wound treatment,^59^ or in pulsatile delivery processes),^71^ the choice could lead towards the use of GAG@ZIF-8, and GAG@MAF-7 biocomposites. However, if a controlled and sustained release of biomolecule is required to reduce the systemic side effects associated with high drug concentrations,^59^ GAG@ZIF-90 biocomposites represent a desirable alternative. As a case study, we examined the encapsulation of HP in GAGs: for HP@MAF-7 we found a loading capacity of 19 wt% and the anticoagulant activity of the released heparin was fully preserved, even after exposure to lyase agents.

Finally, the azolate-based MOF carriers were employed for the encapsulation and release of pre-clinical therapeutics used as anti-inflammatory and anticarcinogenic agents. Similar to the GAG-based biocomposites the EE% and release profiles could be tailored by the judicious selection of the MOF matrix. We anticipate that our findings will facilitate progress in the burgeoning area of MOF-based drug delivery.

## Conflicts of interest

There are no conflicts to declare.

## Supplementary Material

SC-011-D0SC01204A-s001
