# Supplementary material for: Modulation of metal-azolate frameworks for the tunable release of encapsulated glycosaminoglycans
Source: Chem Sci. 2020 Jul 14;11(39):10835–43. doi: 10.1039/d0sc01204a (PMC8162298; doi:10.1039/d0sc01204a)
Supplement: SC-011-D0SC01204A-s001 [file SC-011-D0SC01204A-s001.pdf]

Electronic Supplementary Material (ESI) for Chemical Science.
This journal is © The Royal Society of Chemistry 2020

Electronic Supplementary Information

Modulation of Metal-Azolate Frameworks for the Tunable Release of Encapsulated Glycosaminoglycans

Miriam de J. Velásquez-Hernández [a], Efwita Astria [a], Sarah Winkler [a], Weibin Liang [b], Helmar Wiltsche [c], Arpita Poddar [d], Ravi Shukla [d], Glenn Prestwich [e], John Paderi [f], Pablo Salcedo-Abraira [g], Heinz Amenitsch[h] Patricia Horcajada [g], Christian J. Doonan *[b], and Paolo Falcaro *[a]

E-mail: paolo.falcaro@tugraz.at and christian.doonan@adelaide.edu.au

Abstract: Glycosaminoglycans (GAGs) are biomacromolecules necessary for the regulation of different biological functions. In medicine, GAGs are important commercial therapeutics widely used for the treatment of thrombosis, inflammation, osteoarthritis and wound healing. However, protocols for the encapsulation of GAGs in MOFs carriers are not yet available. Here, we successfully encapsulated GAG-based clinical drugs (heparin, hyaluronic acid, chondroitin sulfate, dermatan sulfate) and two new biotherapeutics in preclinical stage (GM-1111 and HepSYL proteoglycan) in three different pH-responsive metal-azolate frameworks (ZIF-8, ZIF-90, and MAF-7). The resultant GAG@MOF biocomposites present significant differences in terms of crystallinity, particle size, and spatial distribution of the cargo, which influences the drug-release kinetics upon applying an acidic stimulus. For a selected system, heparin@MOF, the released therapeutic retained its antithrombotic activity while the MOF shell effectively protects the drug from Heparin Lyase. By using different MOF shells, the present approach enables the preparation of GAG-based biocomposites with tunable properties such as encapsulation efficiency, protection and release.

Experimental Procedures

General Information

Heparin sodium salt from porcine intestinal mucosa, Hyaluronic acid sodium salt from *Streptococcus equi*, Dermatan sulfate sodium salt from porcine intestinal mucosa, Chondroitin sulfate sodium salt from shark cartilage, FITC-tagged carboxymethyl dextran (FITC-CM-dextran, average Mol. Wt: 40.000, 1-8 mmol FITC/mol glucose, carboxymethyl groups content: 3-7%), and Zinc nitrate hexahydrate ($Zn(NO_3)_2 \cdot 6H_2O$) were purchased from Sigma-Aldrich. 2-Methylimidazole (HmIM), Imidazole-2-carboxylaldehyde (HICA), and 3-methyl-1H-1,2,4-triazole (Hmtz) were purchased from TCI chemicals. Zinc acetate dihydrate $Zn(OAc)_2 \cdot 2H_2O$ and Ammonium sulfamate were purchased from Merck Millipore. Citric acid monohydrate was purchased from Carl Roth. Tri-Sodium citrate dehydrate and Carbazole were purchased from Fluka. Sodium tetraborate was purchased from Honeywell Riedel-de Haën. All reagents and chemicals were used as received without further purification.

FT-IR spectroscopy

FT-IR spectra were recorded on a Bruker ALPHA spectrometer using the ATR accessory with a diamond window in the range of $\tilde{\nu}$ 400 – 4000 cm^{-1}, 128 of scans, resolution 2 cm^{-1}.

Powder X-ray diffraction (PXRD)

PXRD patterns of the samples (GM-1111@ZIF-8, GM-1111@ZIF-90, GM-1111@MAF-7, HepSYL@ZIF-8, HepSYL@ZIF-90 and HepSYL@MAF-7) were recorded on a Rigaku powder diffractometer equipped with D/teX Ultra 250 detector and using Cu Kα radiation (λ = 1.5406 Å). The scan speed was 3 deg min^{-1} and the step 0.01°. WAXS patterns of the samples of (ZIF-8, GAGs@ZIF-8, ZIF-90, GAGs@ZIF-90, MAF-7 and GAGs@MAF-7 were collected at ELETTRA synchrotron using the Austrian SAXS beamline. Operation occurred at a photon energy of 8 keV.

Detector: Pilatus3 100K, Dectris Ltd., Baden, Switzerland; all experiments were performed at room temperature. The resulting two-dimensional images were radially integrated to obtain a 1D pattern of normalized intensity versus scattering vector q. The background was collected using kapton tape and subtracted as background.

Scanning Electron Microscopy (SEM)

SEM images of the samples were recorded by Philips XL30 FEG SEM.

Transmission electron microscopy (TEM)

TEM analysis was carried out in Philips CM100.

Confocal Laser Scanning Microscopy (CLSM)

CLSM data were recorded by Olympus FV3000 microscope, with excitation at 640 nm and emission at 650–675 nm**.**

Determination of Zn by Inductively Coupled plasma-optical emission spectroscopy (ICP-OES)

The Zn concentration was quantified using axially viewed ICP-OES (Ciros Vision EOP, Spectro, Germany) after dilution with 1 mol L^{-1} nitric acid. The Zn (II) 213.856 nm emission line was used.

Thermogravimetric analysis (TGA)

The amount of GAG encapsulated within different materials was assessed by TGA. The thermogravimetric analysis (TGA) was performed using a Perkin Elmer STA 6000 instrument. The program used was of under an air flow (100 mL/min) from 30 ^oC to 800 ^oC with a heating ramp of 5 ^oC/min.

Energy Dispersive X-ray Spectroscopy (EDS) analysis

EDS spectra were collected using Tescan VEGA 3 SEM with tungsten source filament working at 20 kV. Prior the analysis the powder samples were drop casted on a piece of Si (100) treated with a piranha solution to remove traces of organic material. Then, EDS elemental analysis was carried out over areas of 200 x 200 μm^2.The amount of S (wt%) on the free HP was used to determine the contribution of this GAG to the elemental composition of HP@MOFs, whereas the contribution of MOF-shell to the biocomposite was estimated from N (wt%) and Zn (wt%) ratio obtained from the neat MOF.

Synthesis of GAGs@ZIF-8 and GAGs@MAF-7 biocomposites

The synthesis of GAG@ZIF-8 and GAG@MAF-7 biocomposites was carried out using a metal to ligand ratio Zn^{2+}:L = 1:3.47 (L = HmIM, and Hmtz; respectively), and the final concentration of the corresponding biotherapeutic was 0.36 mg mL^{-1}. The stock solution of the corresponding precursors **$Zn(OAc)_2 \cdot 2H_2O$** (80 mM), **2-methylimidazole** (HmIM; 396.6 mM), **3-methyl-1,2,4-triazole** (Hmtz; 396.6 mM), and **GAGs** (2.4 mg mL^{-1}) were prepared in DI water at room temperature. Then, to prepare the GAGs based biocomposites **700 µL** of the required ligand stock solution was premixed with **300 µL** of the corresponding GAG stock solution (or water for control experiments). Finally, **1 mL** of zinc acetate stock solution was added to this mixture. The solutions were left standing at room temperature for 24 h. Afterwards, the solids were collected by centrifugation and washed with deionized water DI (2 mL, 3X), and EtOH (2 mL, 3X). The solids were then air dried at room temperature. Each sample was prepared by triplicate.

Synthesis of GAGs@ZIF-90 biocomposites

The synthesis of GAG@ZIF-90 biocomposites were prepared following a similar procedure described above for GAG@ZIF-8 and GAG@MAF-7 biocomposites, i.e. Zn^{2+}:HICA = 1:3.47; [GAG] = 0.36 mg mL^{-1}. Due to the low solubility of the ligand (HICA) the preparation of the stock solutions was slightly modified. The stock solution of **HICA** (185.1 mM) was prepared in DI water at 60 ^oC under stirring. The stock solutions of **$Zn(NO_3)_2 \cdot 6H_2O$** (400 mM) and the corresponding **GAG** (2.4 mg mL^{-1}) were prepared in DI water at room temperature. To synthesize the GAG@ZIF-90 biocomposites keeping the required metal to ligand ratio (1:3.47) **1.5 mL** of the HICA stock solution at 35 ^oC was premixed with **300 µL** of the corresponding GAG stock solution (or water for control experiments). Then, **200 µL** of **$Zn(NO_3)_2 \cdot 6H_2O$** (400 mM) was added to this mixture. The solutions were left standing at room temperature for 24 h. Afterwards, the solids were collected by centrifugation and washed with deionized water DI (2 mL, 3X), and EtOH (2 mL, 3X). The solids were then air dried at room temperature. Each sample was prepared by triplicate.

General Procedure for Encapsulation Efficiency measurement

The encapsulation efficiency (EE%) of each GAG@MOF biocomposite was assessed using UV-vis spectroscopy and the carbazole assay, which is a direct method to quantify glycosaminoglycans by colorimetry (λ_{max} 520 nm) (*vide infra*). To determine the EE% of GAG@MOF biocomposites, each sample was soaked in citrate buffer (2 mL, 80 mM, pH = 6). The clear solution obtained was filtered through a size exclusion chromatography column to remove the ligand and Zn^{2+} ions released during the degradation

process. This improves the determination of the amount of GAG in the solution by reducing the interference of the degraded MOF components and the buffer media with the carbazole assay.

Carbazole assay

Ammonium sulfamate (20 µL, 4 M) was added to an aliquot of sample (200 µL) or water (blank control) and the resultant mixture was vortexed for 1 min. Then, sodium tetraborate in sulfuric acid (1 mL, 25 mM) was added and carefully mixed. The mixture was heated at 100 ^oC for 5 min and cooled to room temperature. Afterwards, the carbazole solution (0.1%, 40 µL) was added and the resultant mixture was heated again at 100 ^oC for 15 min and then cooled down to room temperature (color develops during this step). Finally, the resultant solution was analyzed by UV-vis spectroscopy; the absorbance at 520 nm was used to quantify the amount of the analyte by comparison with the corresponding calibration curve (Fig. S23). All the experiments were performed in triplicate.

Release test of GAG@MOFs

1 mL of citrate buffer (80 mM, pH = 6) was added to a pellet of GAGs@MOF, the sample was kept under bidimensional stirring. Aliquots of 200 µL of the supernatant were collected by centrifugation (13400 rpm, 1 min) and replaced with the same volume of fresh medium. The amount of GAG released in the incubation media was the determined by UV-vis spectroscopy using carbazole assay.

Preliminary studies for determining the synthetic conditions to optimize the encapsulation efficiency (EE%)

Synthesis of FITC-CMD@ZIF-8 and FITC-CMD@MAF-7 biocomposites

The synthesis of ZIF-8 and MAF-7 based biocomposites were carried out using three different Zn^{2+}:L (L = HmIM, and Hmtz; respectively) ratios: 1:4 (**A**), 1:3.47 (**B**) and 1:2.52 (**C**). For each ratio, we used five different concentrations of FITC-CM-dextran (FITC-CMD) : 0 (**1**), 0.18 (**2**), 0.36 (**3**), 0.72 (**4**), 1.44 (**5**) mg mL^{-1} (**Table S1**). The stock solution of the corresponding precursors **$Zn(OAc)_2·2H_2O$** (80 mM), **2-Methylimidazole** (HmIM; 800 mM), **3-methyl-1,2,4-triazole** (Hmtz; 800 mM), and **FITC-CM-dextran** (5.76 mg mL^{-1}) were prepared in DI water at room temperature. To synthesize the samples FITC-CMD@ZIF-8 (**8DXAn**, **8DXBn**, **and 8DXCn**; **n** = **1–5**) and FITC-CMD@MAF-7 (**7DXAn**, **7DXBn**, **and 7DXCn**; **n** = **1–5**), the required volume of the corresponding ligand (HmIM or Hmtz), and FITC-CMD stock solutions were premixed reaching a total volume of 1 mL, by dilution with water. Then, 1 mL of $Zn(OAc)_2·2H_2O$ was added to this mixture (**Table S1**). The resultant mixtures were left standing at room temperature for 24 h. Afterwards, the solids were collected by centrifugation and washed with deionized water DI (2 mL, 3X), and EtOH (2 mL, 3X). The solids were then air-dried at room temperature. Each sample was prepared by triplicate.

Sample	Ratio	Final concentration			Volume (µL)			
	M:L	L (mM)	CM-DEXT (mg mL^{-1})	$Zn(OAc)_2·2H_2O$ (mM)	L	CM-DEXT	H_2O	$Zn(OAc)_2·2H_2O$
**nDXA1**	1:4	160	0	40	400	0	600	1000
**nDXA2**	1:4	160	0.18	40	400	62.5	537.5	1000
**nDXA3**	1:4	160	0.36	40	400	125	475	1000
**nDXA4**	1:4	160	0.72	40	400	250	350	1000
**nDXA5**	1:4	160	1.44	40	400	500	100	1000
**nDXB1**	1:3.47	138.8	0	40	347	0	653	1000
**nDXB2**	1:3.47	138.8	0.18	40	347	62.5	590.5	1000
**nDXB3**	1:3.47	138.8	0.36	40	347	125	528	1000
**nDXB4**	1:3.47	138.8	0.72	40	347	250	403	1000
**nDXB5**	1:3.47	138.8	1.44	40	347	500	153	1000
**nDXC1**	1:2.52	100.8	0	40	252	0	748	1000
**nDXC2**	1:2.52	100.8	0.18	40	252	62.5	685.5	1000
**nDXC3**	1:2.52	100.8	0.36	40	252	125	623	1000
**nDXC4**	1:2.52	100.8	0.72	40	252	250	498	1000
**nDXC5**	1:2.52	100.8	1.44	40	252	500	248	1000

Table S1. Synthetic protocol for FITC-CMD@ZIF-8 (**n** = **8**) and FITC-CMD@MAF-7 biocomposites (**n** = **7**) biocomposites.

Synthesis of FITC-CMD@ZIF-90

The synthesis of ZIF-90 based biocomposites was carried out using three different Zn^{2+}: HICA ratios: 1:4 (**A**), 1:3.47 (**B**) and 1:2.52 (**C**). For each ratio, we used five different concentrations of FITC-CMD: 0 (**1**), 0.18 (**2**), 0.36 (**3**), 0.72 (**4**), 1.44 (**5**) mg mL^{-1} (**Table S2**). The stock solutions of **2-Imidazolecarboxaldehyde** (HICA) (213.33 mM (for ratio **A**), 185.1 mM (for ratio **B**) and 134.4 mM (for ratio **C**)) were prepared in DI water at 60 °C. Whereas the stock solutions of **$Zn(NO_3)_2·6H_2O$** (320 mM) and **FITC-CM-dextran** (S1 = 11.52 mg mL^{-1}, and 2.88 mg mL^{-1}) were prepared in DI water at room temperature. To synthesize the samples **90DXAn**, **90DXBn**, and **90DXCn** (**n** = **1** – **5**) the required volume of each stock solution was mixed according to **Table S2**. The resultant mixtures were left standing at room temperature for 24 h. Afterwards, the solids were collected by centrifugation and washed with deionized water DI (2 mL, 3X), and EtOH (2 mL, 3X). The solids were then air dried at room temperature. Each sample was prepared by triplicate.

Sample	Ratio	Final concentration			Volume (mL)			
	M:L	HICA (mM)	FITC-CMD (mg mL^{-1})	$Zn(NO_3)_2·6H_2O$ (mM)	HICA	FITC-CMD	H_2O	$Zn(NO_3)_2·6H_2O$
**90DXA1**	1:4	160	0	40	1.5	0	0.25	0.25
**90DXA2**	1:4	160	0.18	40	1.5	0.125 (S2)	0.125	0.25
**90DXA3**	1:4	160	0.36	40	1.5	0.250 (S2)	0	0.25
**90DXA4**	1:4	160	0.72	40	1.5	0.125 (S1)	0.125	0.25
**90DXA5**	1:4	160	1.44	40	1.5	0.250 (S1)	0.250	0.25
**90DXB1**	1:3.47	138.8	0	40	1.5	0	0.25	0.25
**90DXB2**	1:3.47	138.8	0.18	40	1.5	0.125 (S2)	0.125	0.25
**90DXB3**	1:3.47	138.8	0.36	40	1.5	0.250 (S2)	0	0.25
**90DXB4**	1:3.47	138.8	0.72	40	1.5	0.125 (S1)	0.125	0.25
**90DXB5**	1:3.47	138.8	1.44	40	1.5	0.250 (S1)	0.250	0.25
**90DXC1**	1:2.52	100.8	0	40	1.5	0	0.25	0.25
**90DXC2**	1:2.52	100.8	0.18	40	1.5	0.125 (S2)	0.125	0.25
**90DXC3**	1:2.52	100.8	0.36	40	1.5	0.250 (S2)	0	0.25
**90DXC4**	1:2.52	100.8	0.72	40	1.5	0.125 (S1)	0.125	0.25
**90DXC5**	1:2.52	100.8	1.44	40	1.5	0.250 (S1)	0.250	0.25

Table S2. Synthetic protocol for FITC-CMD@ZIF-90 biocomposites.

Determination of the encapsulation efficiency of FITC-CMD@ZIF-8, FITC-CMD@ZIF-90, and FITC-CMD@MAF-7 biocomposites

The quantitative assessment of the cargo loaded within the biocomposites was carried out by re-dissolving the MOF matrix under acidic conditions, soaking the samples in 2 mL of citrate buffer (100 mM, pH = 6). The resultant solution was analyzed by UV-vis spectroscopy, where the absorbance at 490 nm was used to quantify the amount of the analyte by comparison with the corresponding calibration curve (Fig. S1-S3). To avoid any interference of the ligand, metal, and/or the citrate buffer during the determination process, the calibration curves were performed adding a known amount of FITC-CM-dextran to a solution of Zn^{2+} and the corresponding ligand (HICA or Hmtz) in citrate buffer media. The amount of Zn^{2+} and ligand added to this mixture depends of the amount of material formed for each metal to ligand ratio. These experiments were performed in triplicate for each sample described in the Tables S1 and S2.

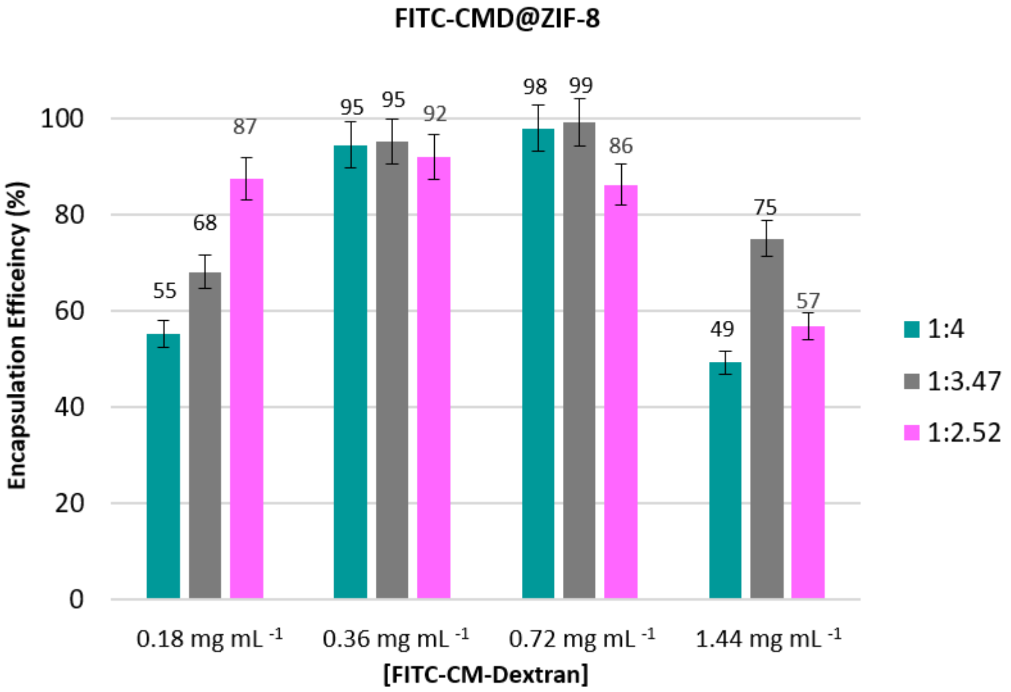

Fig. S1 Encapsulation efficiency of FITC-CMD@ZIF-8 biocomposites obtained varying the Zn^{2+}:HmIM ratio and the concentration of FITC-CMD.

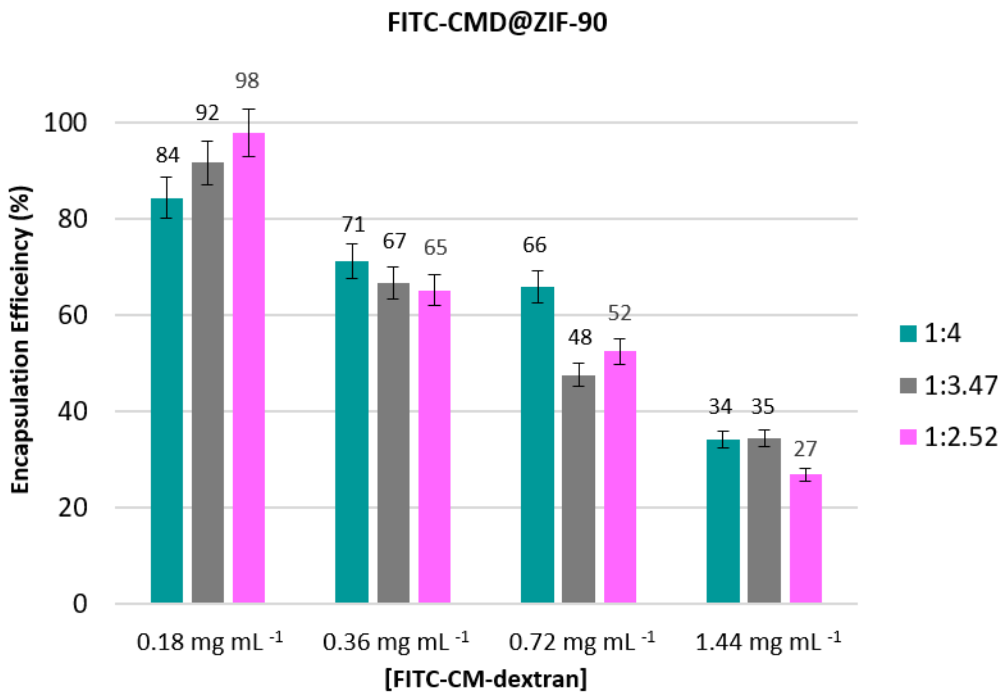

Fig. S2 Encapsulation Efficiency of FITC-CMD@ZIF-90 biocomposites obtained varying the Zn^{2+}:HICA ratio and the concentration of FITC-CMD.

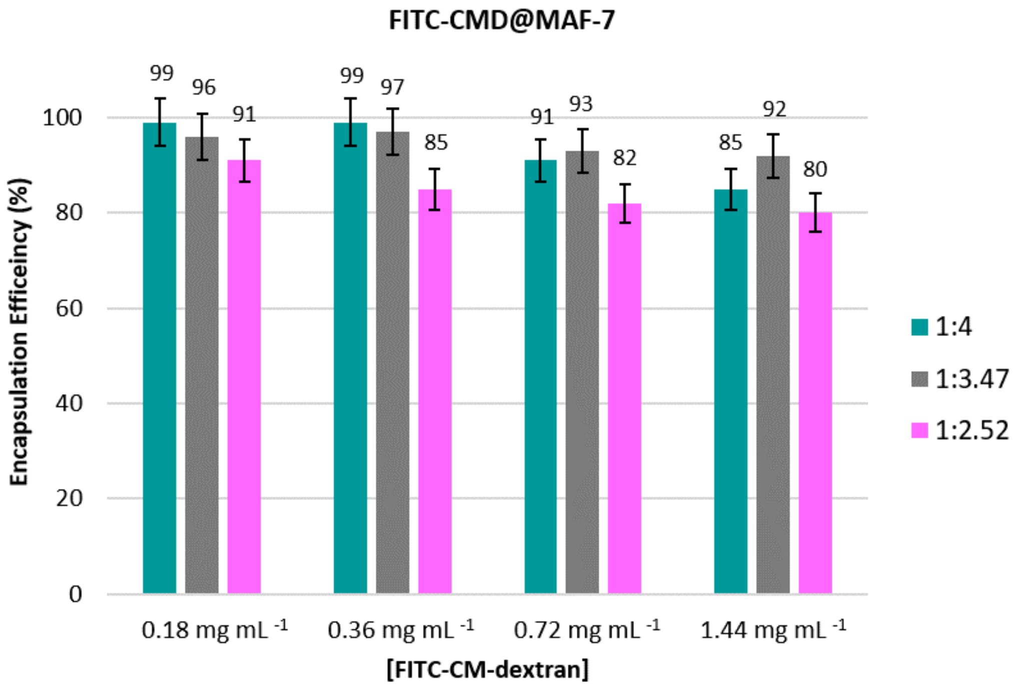

Fig. S3 Encapsulation Efficiency of FITC-CMD@MAF-7 biocomposites obtained varying the Zn^{2+}:Hmtz ratio and the concentration of FITC-CMD.

Results reveal that the FITC-CMD@ZIF-8 biocomposites obtained from 0.36 (**3**) and 0.72 (**4**) mg mL^{-1} of FITC-CMD present higher EE% than those obtained from 0.18 (**2**) and 1.44 (**5**) mg mL^{-1} of FITC-CMD (Fig. S1). In addition, the EE% is influenced by the metal to ligand ratio: the optimal encapsulation efficiency is reached when using 0.36 (**3**) and 0.72 (**4**) mg mL^{-1} of FITC-CMD and 1:4 and 1:3.47 metal to ligand ratio (**8DXA3**, **8DXA4**, **8DXB3**, and **8DXB4**). for FITC-CMD@ZIF-90 biocomposites (**90DXAn**; where **n** = **2** – **5**), the EE% decreases drastically with the increase in the initial concentration of FITC-CMD from *ca.* 84% for **90DXA2** to *ca.* 34% for **90DXA5** (Fig. S2). Such results suggest that the EE% strongly depends on the initial concentration of the model drug.

FITC-CMD@MAF-7 biocomposites obtained from 1:4 metal to ligand ratio (**7DXAn**; where **n** = **2** – **5**) present exceptional polysaccharide payloads regardless of the initial concentration of FITC-CMD. For instance, the EE% is almost quantitative for the samples prepared with low initial concentrations of FITC-CMD (EE > 90% for **7DXA2**, **7DXA3**, and **7DXA4**). The EE% decreases slightly as the concentration of FITC-CMD increases (*ca.* 85% for **7DXA5**) (Fig. S3). These findings are consistent with the previous reports about the biomineralization of carbohydrates within ZIF-8.[1] However, such reports also declare that Zn^{2+}:L ratio affects the polysaccharide payloads. Thus, in concordance with these studies, another two different metal to ligand ratios were tested (Zn^{2+}:L = 1:3.47 (**B**) and 1:2.52 (**C**)) to corroborate the role of this parameter in the EE of the resultant FITC-CMD@MAF-7 and FITC-CM-dextran@ZIF-90 biocomposites (**7DXBn**, **7DXCn**, **90DXBn**, and **90DXCn**; where **n** = **2** – **5**) (Table S1 and S2). The collected data indicate that high polysaccharide payloads were achieved in FITC-CMD@MAF-7 biocomposites when using a 1:3.47 ratio (EE > 90%) regardless of the concentration of FITC-CM-dextran (Fig. S3). Whereas, the biocomposites obtained from Zn^{2+}:L = 1:2.52 ratio present lower EE than their analogous derived from Zn^{2+}:L = 1:3.47 and 1:4 (Fig. S3). Regarding FITC-CMD@ZIF-90 biocomposites, the trend of EE because of the variations in the Zn^{2+}:L ratio is not as clear as for MAF-7 biocomposites. Since for ZIF-90-based biocomposites a synergistic effect between Zn^{2+}:L ratio and the initial concentration of FITC-CMD determines the final EE (Fig. S3).

Release test of FITC-CMD@ZIF-8 FITC-CMD@MAF-7 and FITC-CMD@ZIF-90

The drug release performance of the resultant MAF-7 and ZIF-90-based biocomposites (**8DXAn**, **8DXBn**, **8DXCn**, **7DXAn**, **7DXBn**, **7DXCn** and **90DXAn**, **90DXBn**, and **90DXCn**; where **n** = **2** – **5**) was assessed by monitoring the amount of FITC-CM-dextran released over time upon applying an external acidic stimulus (Fig. S4–S7). Thus, the powder material was soaked in 1 mL of citrate buffer (100 mM, pH = 6) under bidimensional continuous stirring (500 rpm). At different incubation times the sample was centrifuged, and 1 mL of the supernatant was taken to be analyzed by UV-vis spectroscopy (λ_{max} 490 nm). It is worth to mention that the samples prepared with MAF-7 keeping Zn^{2+}:L = 1:2.52 ratio degrades almost immediately upon the addition of citrate buffer.

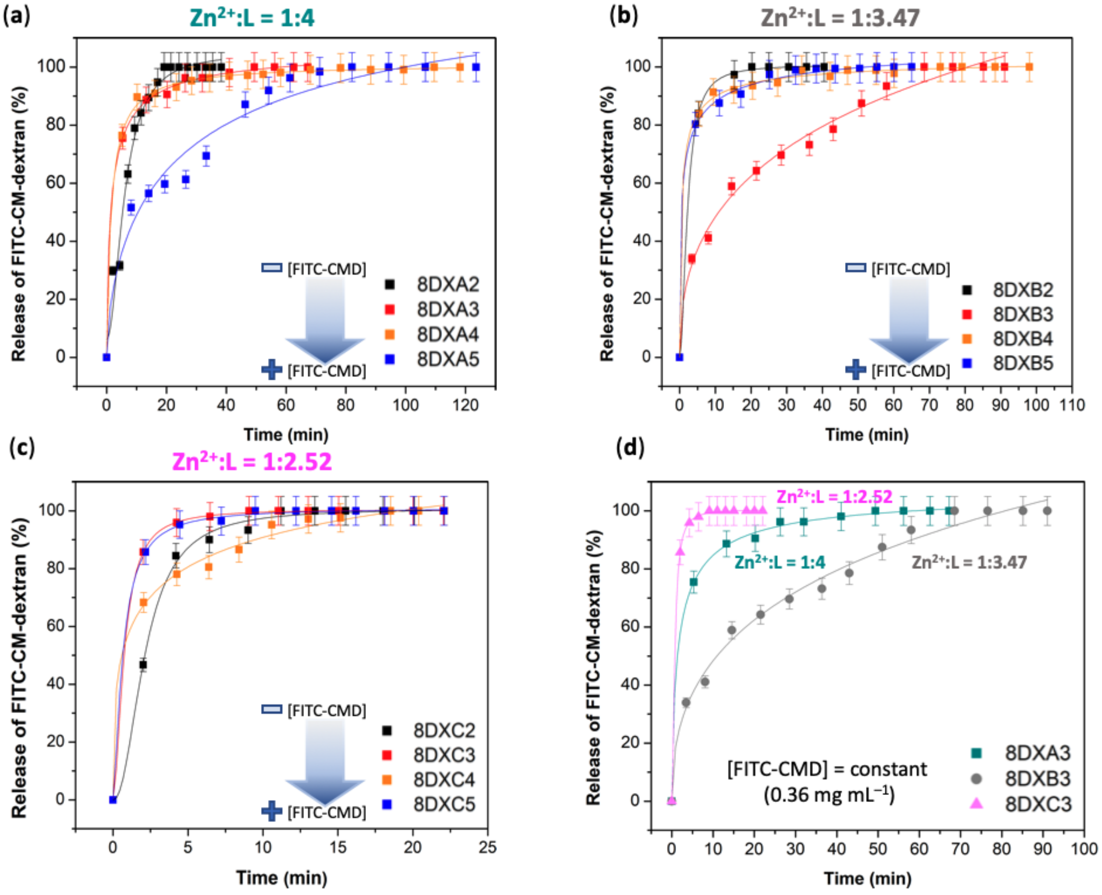

Fig. S4. Release profiles of the FITC-CMD@ZIF-8 biocomposites. from: (a) Zn^{2+}:HmIM =1:4, (b) Zn^{2+}: HmIM =1:3.47, (c) Zn^{2+}: HmIM =1:2.52 varying the initial concentration of FITC-CMD. (d) Comparative release profiles of samples obtained from different Zn^{2+}: HmIM ratios keeping constant the initial concentration of FITC-CMD (0.36 mg mL^{-1}).

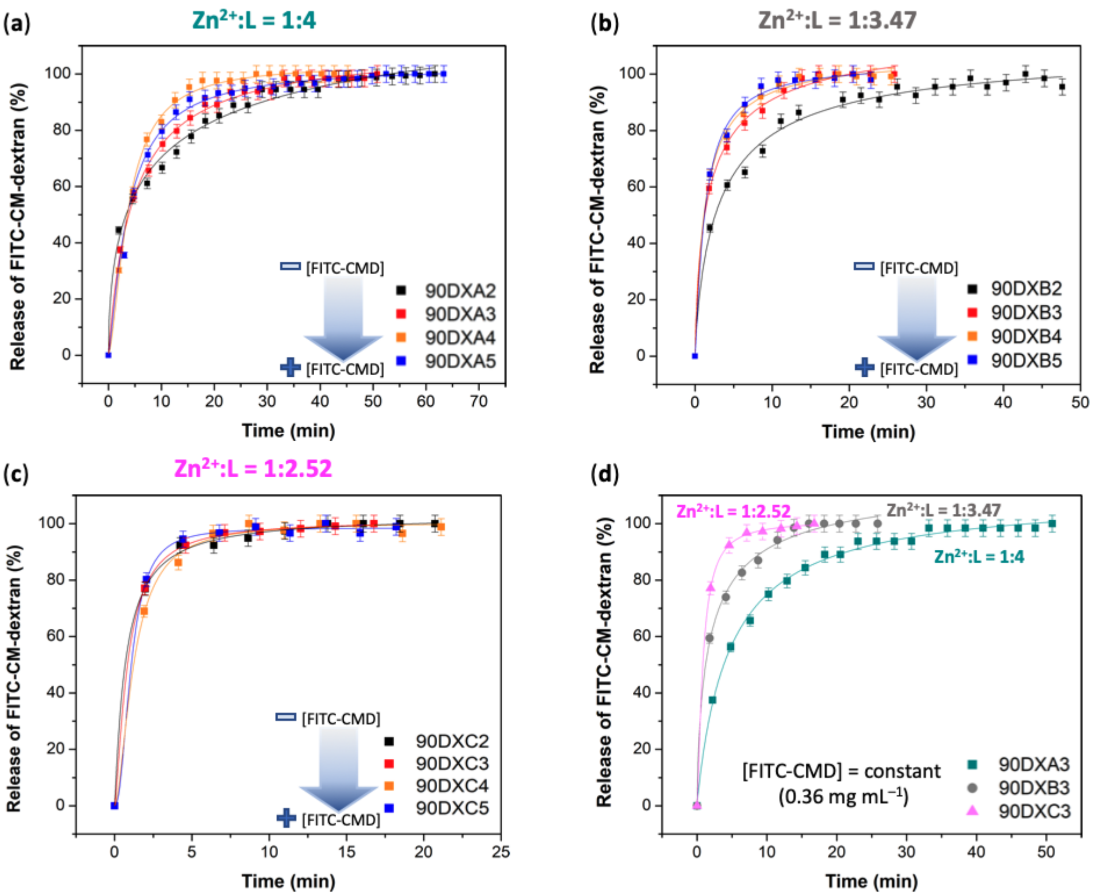

Fig. S5. Release profiles of the FITC-CMD@ZIF-90 biocomposites. from: (a) Zn^{2+}:HICA=1:4, (b) Zn^{2+}:HICA=1:3.47, (c) Zn^{2+}:HICA=1:2.52 varying the initial concentration of FITC-CMD. (d) Comparative release profiles of samples obtained from different Zn^{2+}:HICA ratios keeping constant the initial concentration of FITC-CMD (0.36 mg mL^{-1}).

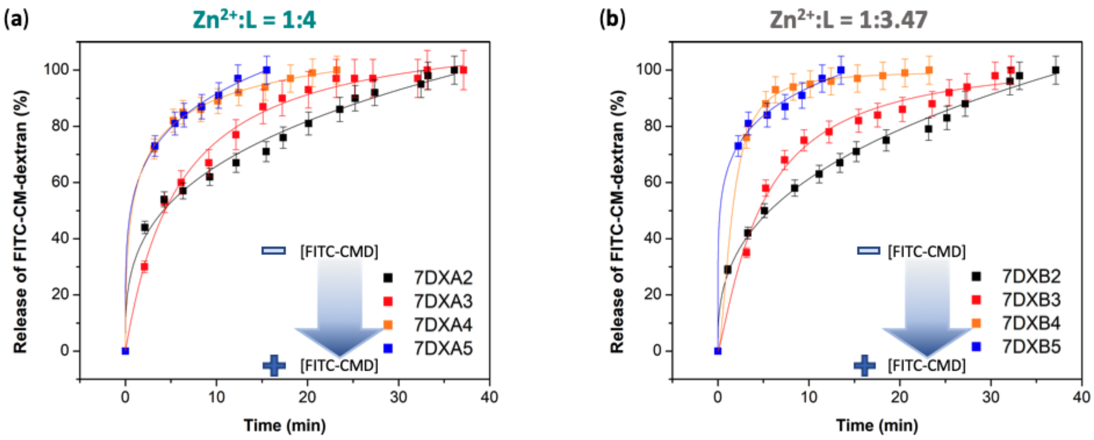

Fig. S6. Release profiles of the FITC-CMD@MAF-7 biocomposites. (a) from Zn^{2+}:Hmtz=1:4 ratio varying the initial concentration of FITC-CMD, and (b) from Zn^{2+}:Hmtz=1:3.47 varying the initial concentration of FITC-CMD.

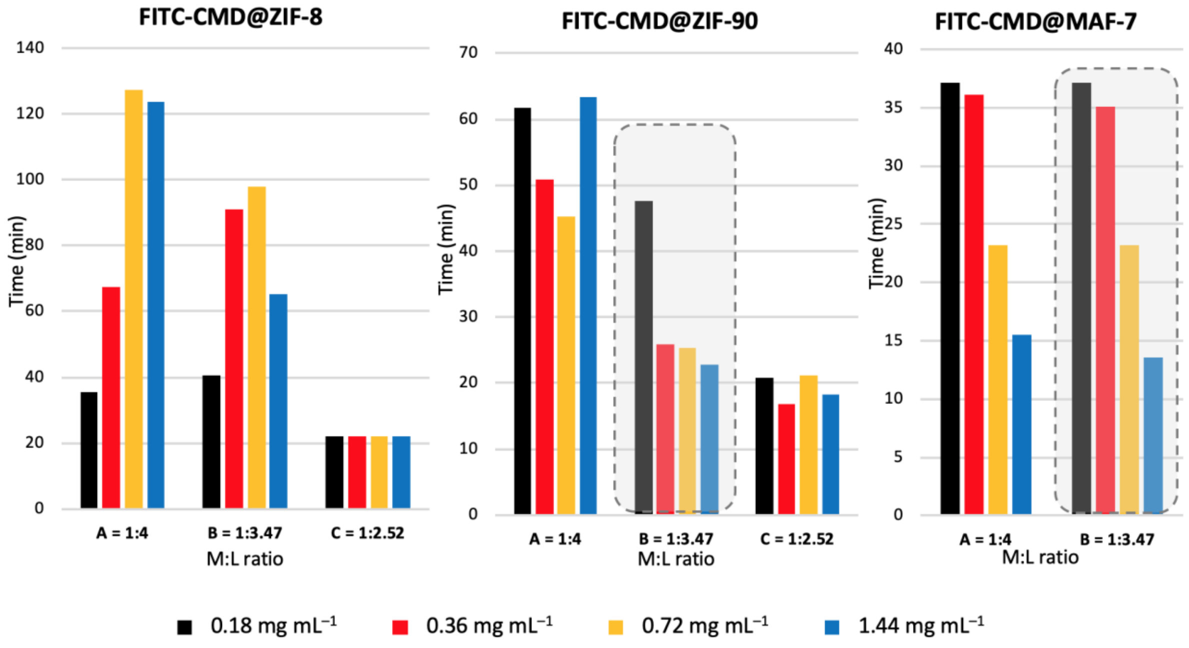

Fig. S7. Time required to release 100% of FITC-CMD from the MOF biocomposites obtained by varying the initial concentration of the model drug (FITC-CMD) and the Zn^{2+}:L ratio.

In general, the release profiles of ZIF-8, ZIF-90 and MAF-7 biocomposites present an initial burst release, followed by a sustained delivery process (Fig. S4–S7).

After a close inspection of the release profiles obtained from different metal to ligand ratio, it is evident that the delivery rate increases as the Zn^{2+}:L decreases. For instance, a comparative analysis of the release kinetics obtained from **90DXA3**, **90DXB3**, and **90DXC3** (Zn^{2+}:L = 1:4, 1:3.47, and 1:2.52; respectively) reveals that **90DXA3** required around 50 min to achieve the full release of the model drug; whereas its analogous **90DXB3** and **90DXC3** achieved the complete release of the cargo within 25 and 15 min, respectively (Fig. S5). Similarly, for MAF-7-based biocomposites, it was observed that the samples obtained from Zn^{2+}:L= 1:2.52 (**7DXCn**) degraded immediately upon soaking them into the acidic media. By contrast, for those prepared from 1:4 (**A**) and 1:3.47 (**B**) Zn^{2+}:L ratio took from 15 min (**7DXA5** and **7DXB5**) to 30 min (**7DXA2**, **7DXA3**, **7DXB2** and **7DXB3**) to achieve the full release of the cargo (Fig. S6).

In light of such findings, we conclude that the optimal synthetic conditions to ensure acceptable encapsulation efficiencies and drug release kinetics, for all the three different metal-azolate systems, requires the usage of 0.36 mg mL^{-1} of the biomacromolecule keeping the ratio Zn^{2+}:L = 1:3.47.

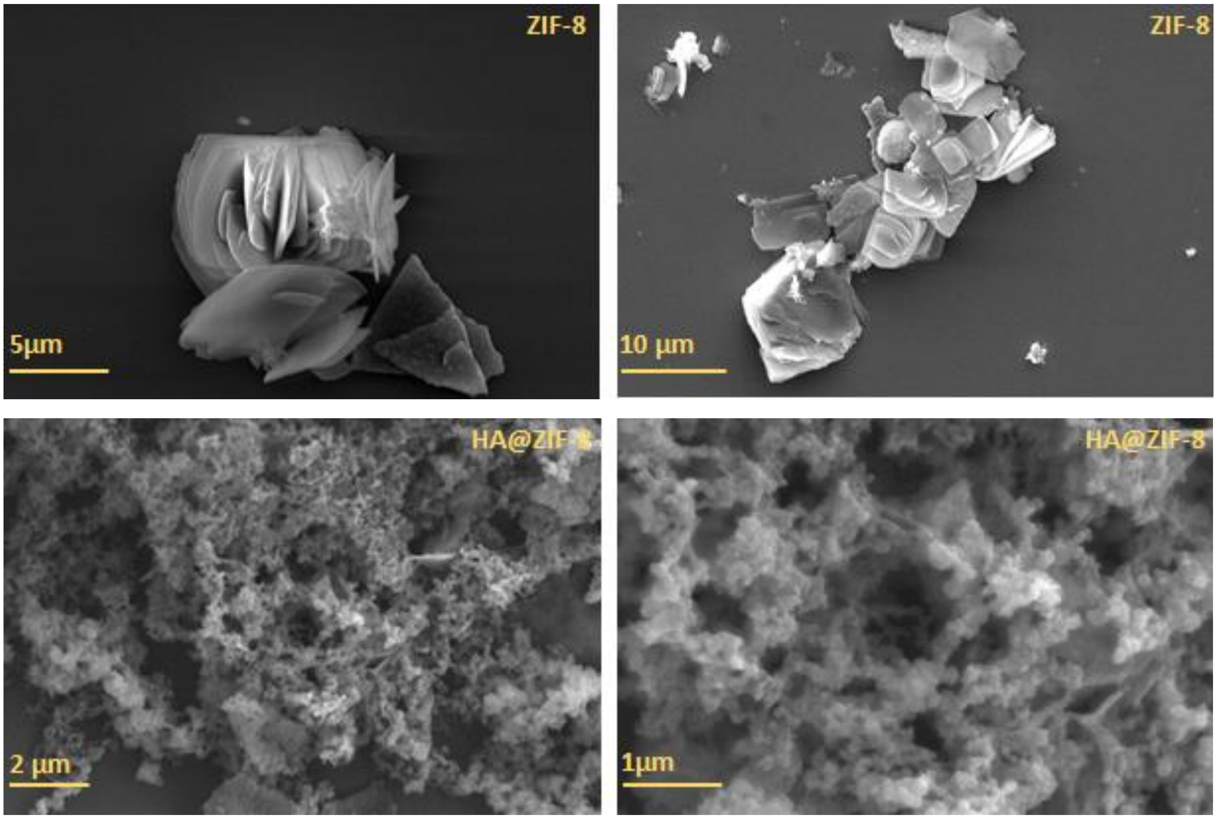

Fig. S8 SEM images for ZIF-8 and HA@ZIF-8.

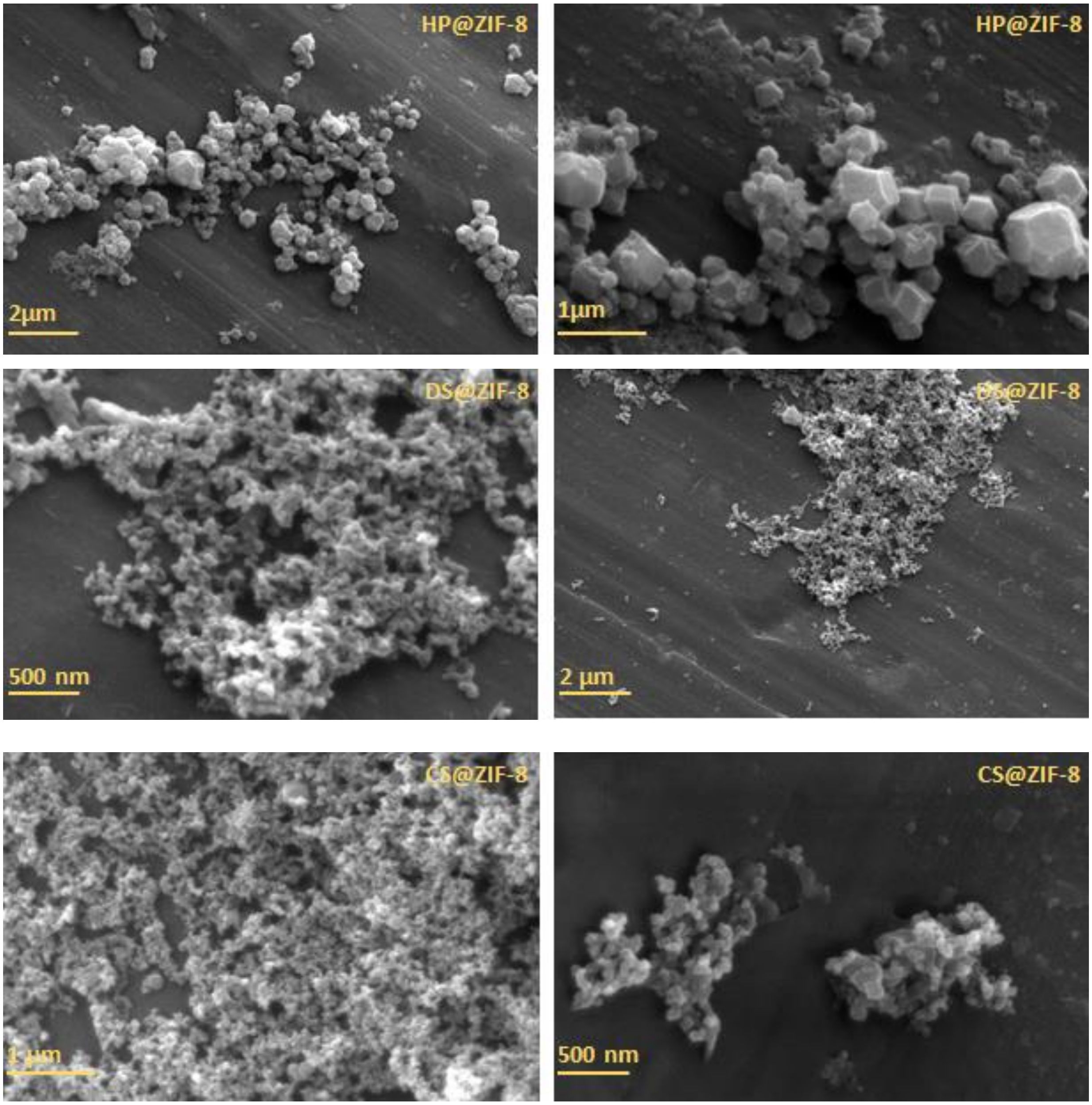

Fig. S9 SEM images for HP@ZIF-8, DS@ZIF-8, and CS@ZIF-8.

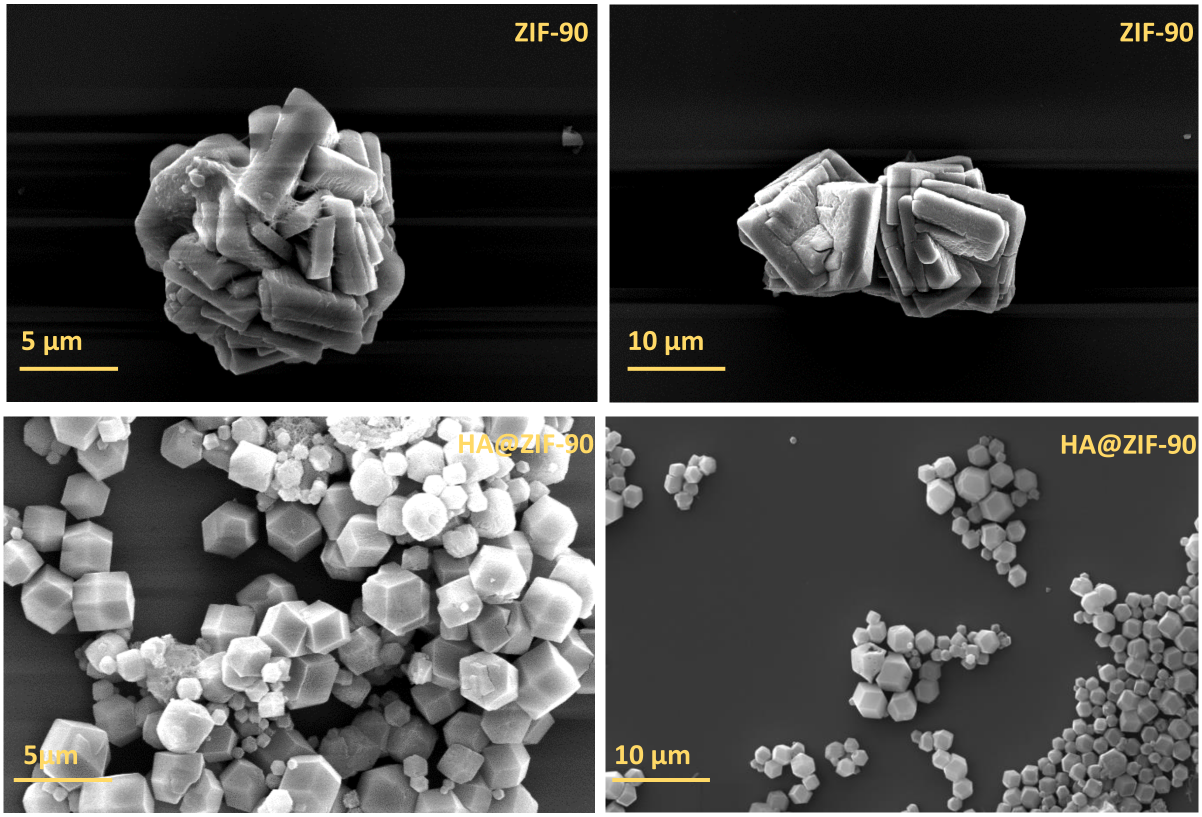

Fig. S10 SEM images of pure ZIF-90 and HA@ZIF-90.

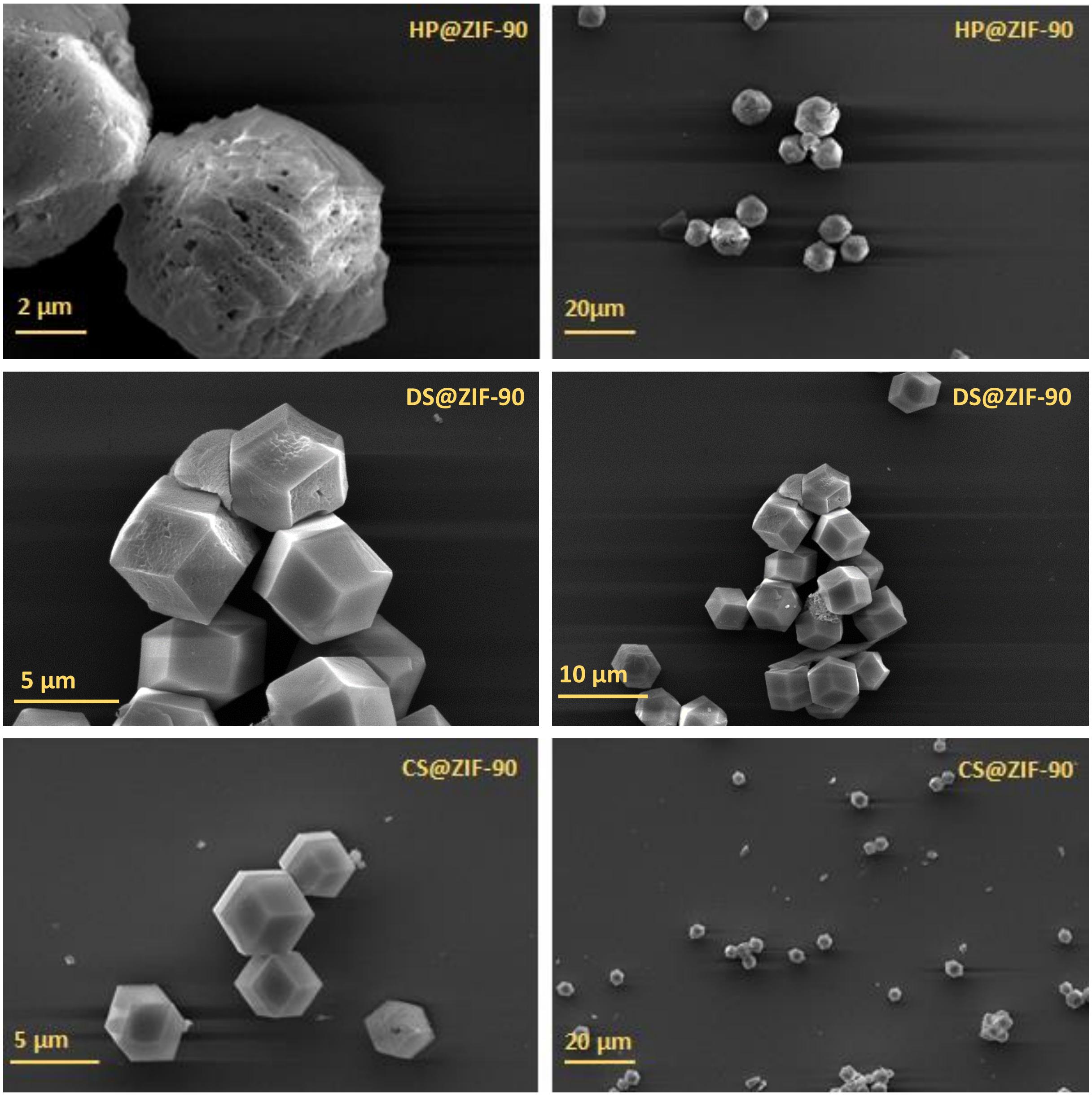

Fig. S11 SEM images for HP@ZIF-90, CS@ZIF-90, and DS@ZIF-90.

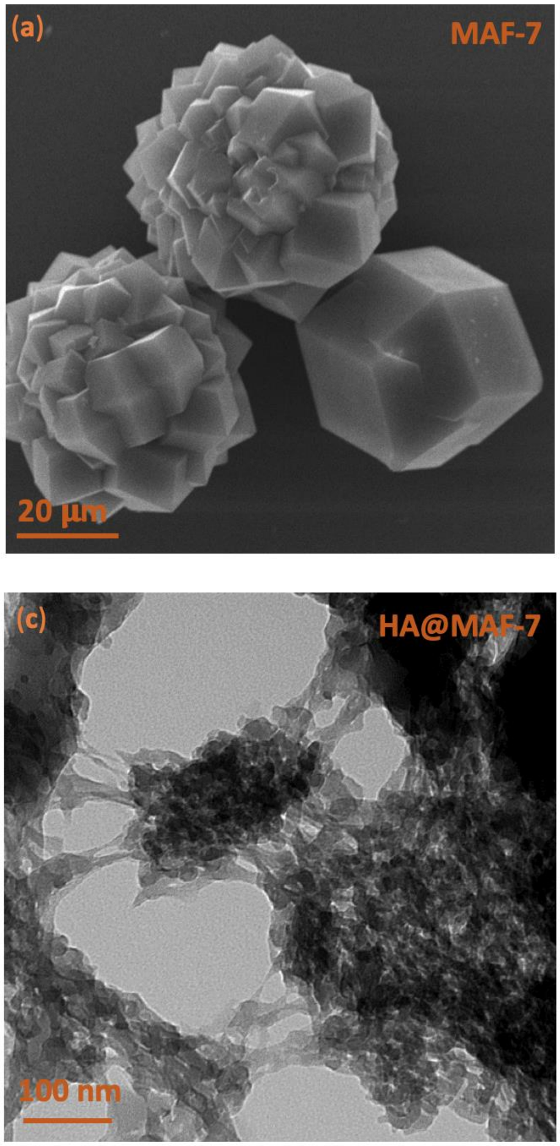

Fig. S12. (a) SEM image of MAF-7 synthesized in presence of $NH_3 \cdot H_2O$ (10 %). (b) TEM image of MAF-7 synthesized without ammonia. (c) and (d) TEM images of HA@MAF-7.

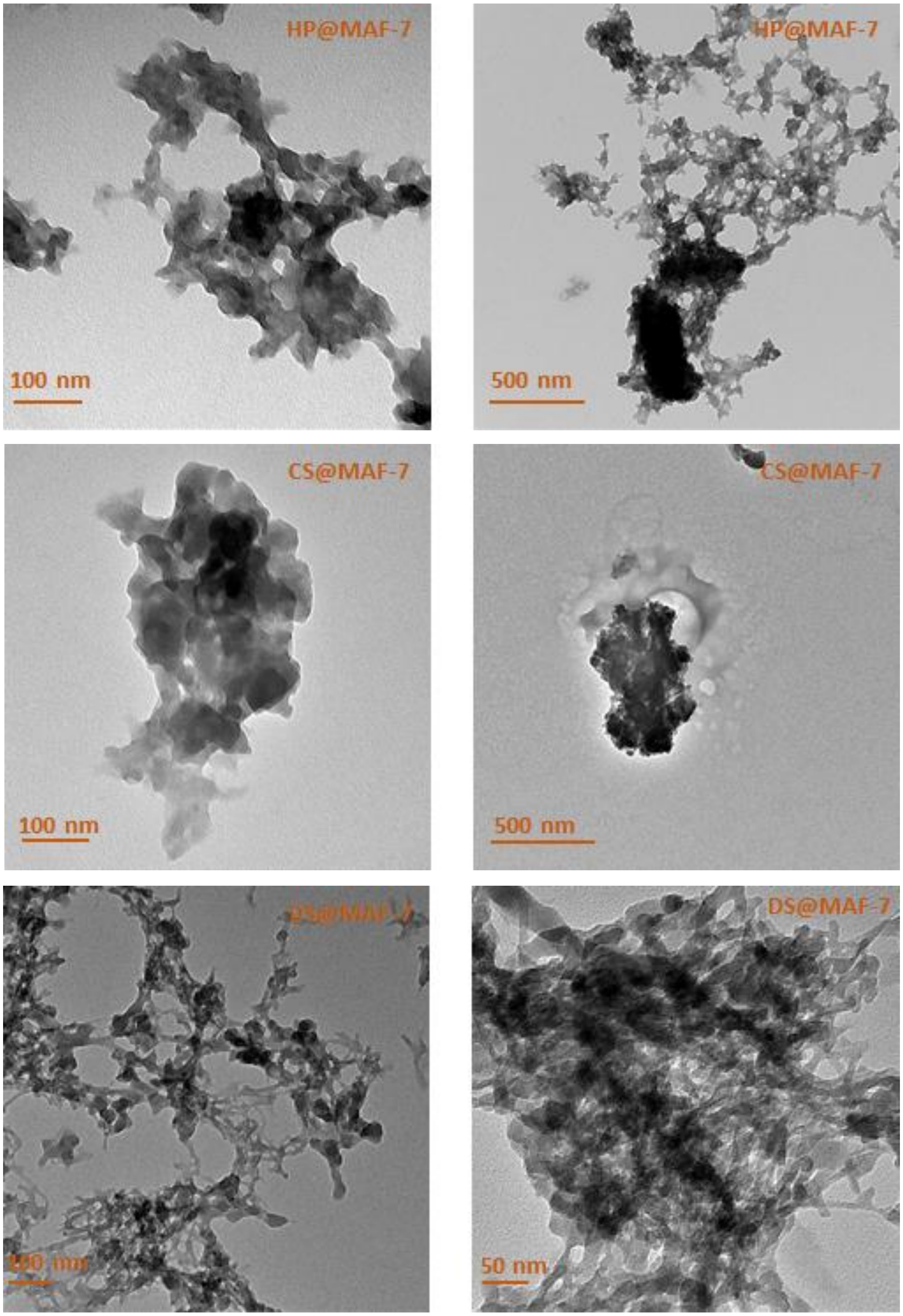

Fig. S13 TEM images for HP@MAF-7, CS@MAF-7, and DS@MAF-7.

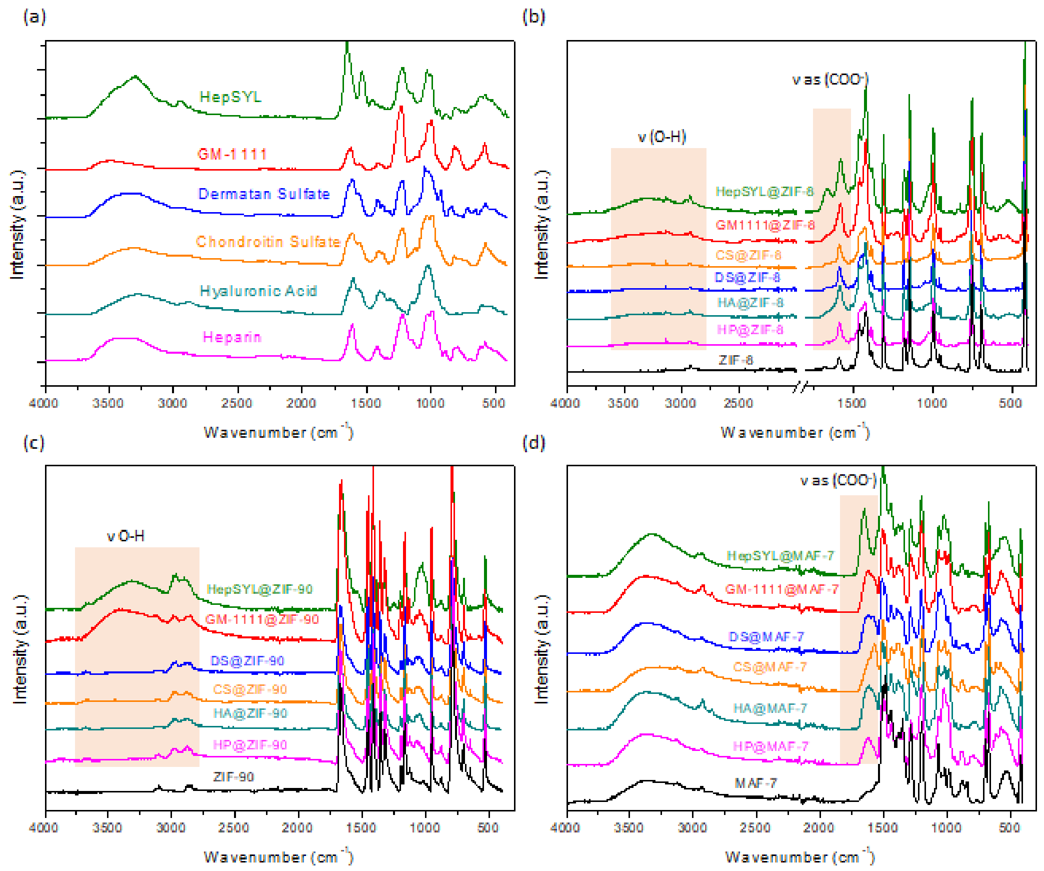

Fig. S14 FTIR spectra of GAGs and GAGs@MOFs (a) FTIR spectra of GAGs. (b) FTIR spectra of GAGs@ZIF-8. (c) FTIR spectra of GAGs@MAF-7 (d) FTIR spectra of GAGs@ZIF-90.

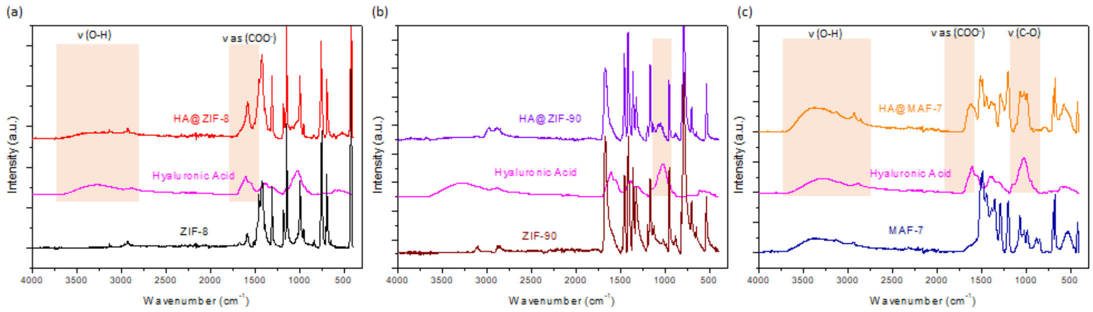

Fig. S15 FTIR spectra of HA@MOFs (a) FTIR spectra of HA@ZIF-8 (b) FTIR spectra HA@ZIF-90 of (c) FTIR spectra HA@MAF-7

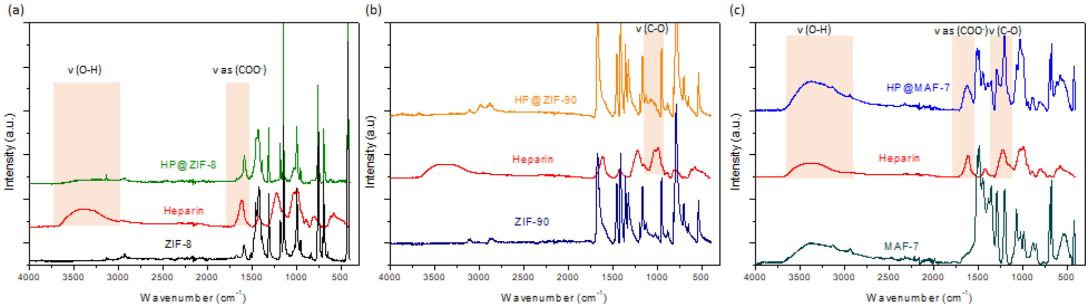

Fig. S16 FTIR spectra of HP@MOFs**.** (a) FTIR spectra of HP@ZIF-8. (b) FTIR spectra of HP@ZIF-90 (c) FTIR spectra HP@MAF-7.

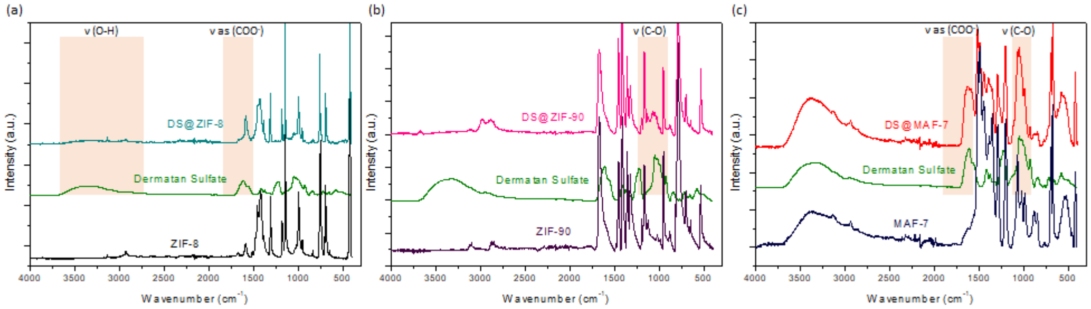

Fig. S17 FTIR spectra of DS@MOFs (a) FTIR spectra of DS@ZIF-8 (b) FTIR spectra of DS@ZIF-90 (c) FTIR spectra DS@MAF-7.

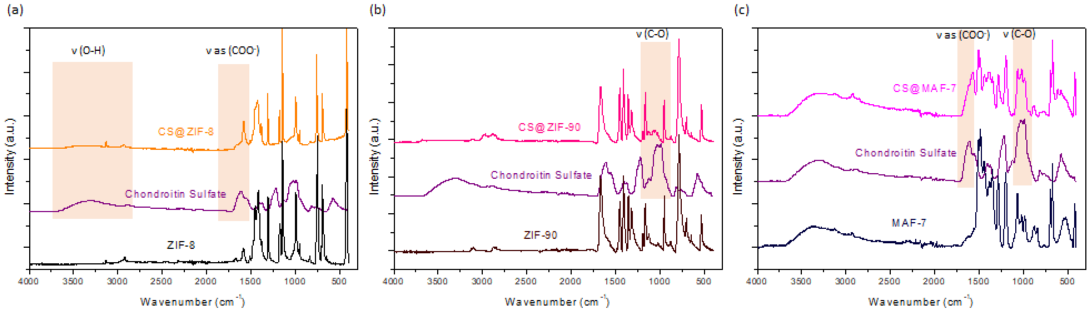

Fig. S18 FTIR spectra of CS@MOFs (a) FTIR spectra of CS@ZIF-8 (b) FTIR spectra of CS@ZIF-90 (c) FTIR spectra CS@MAF-7.

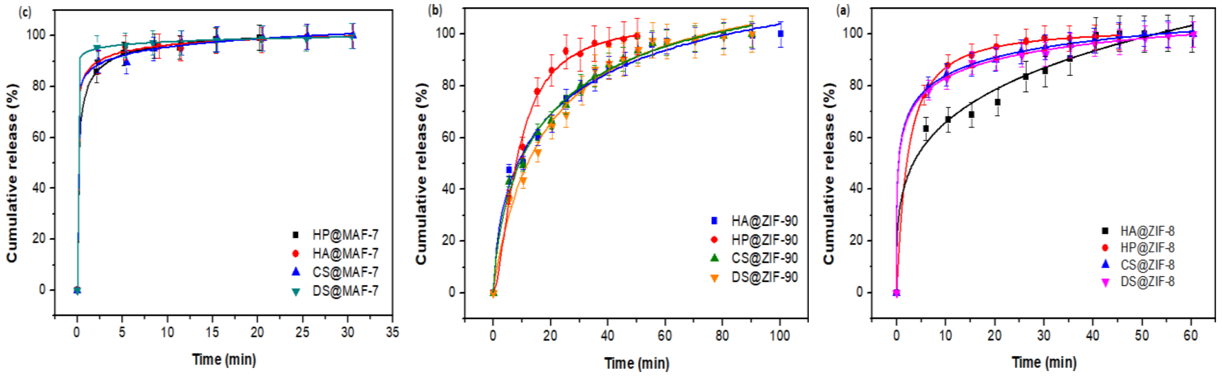

Fig. S19 Drug release kinetics of GAG@MOFs biocomposites. (a) Drug release profile of GAG@MAF-7; (b) Drug release profile of GAG@ZIF-90; (c) Drug release profile of GAG@ZIF-8.

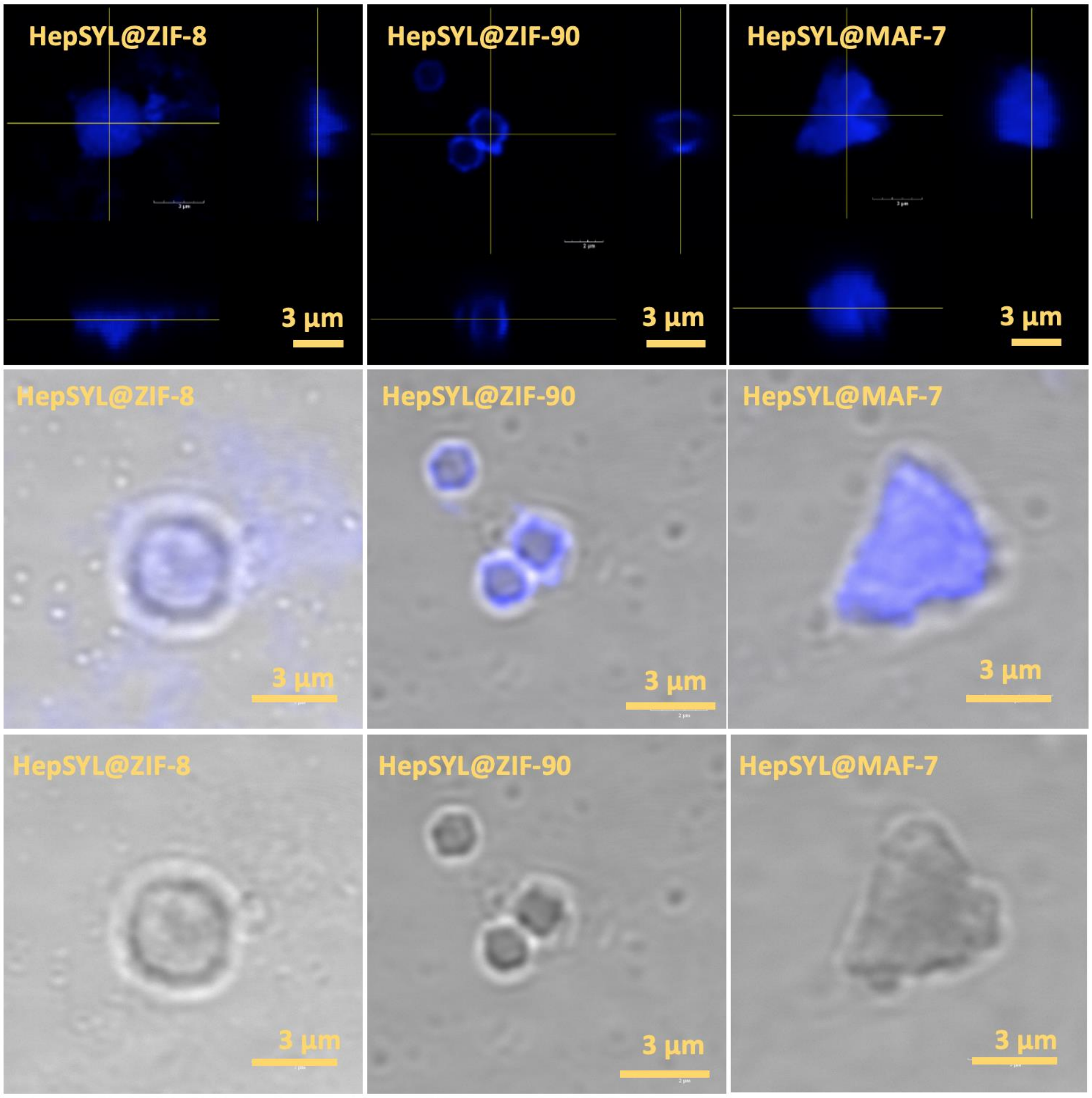

Fig. S20. Confocal laser scanning micrographs showing the fluorescence, overlay and bright field, images of HepSYL@ZIF-8, HepSYL@ZIF-90, and HepSYL@MAF-7.

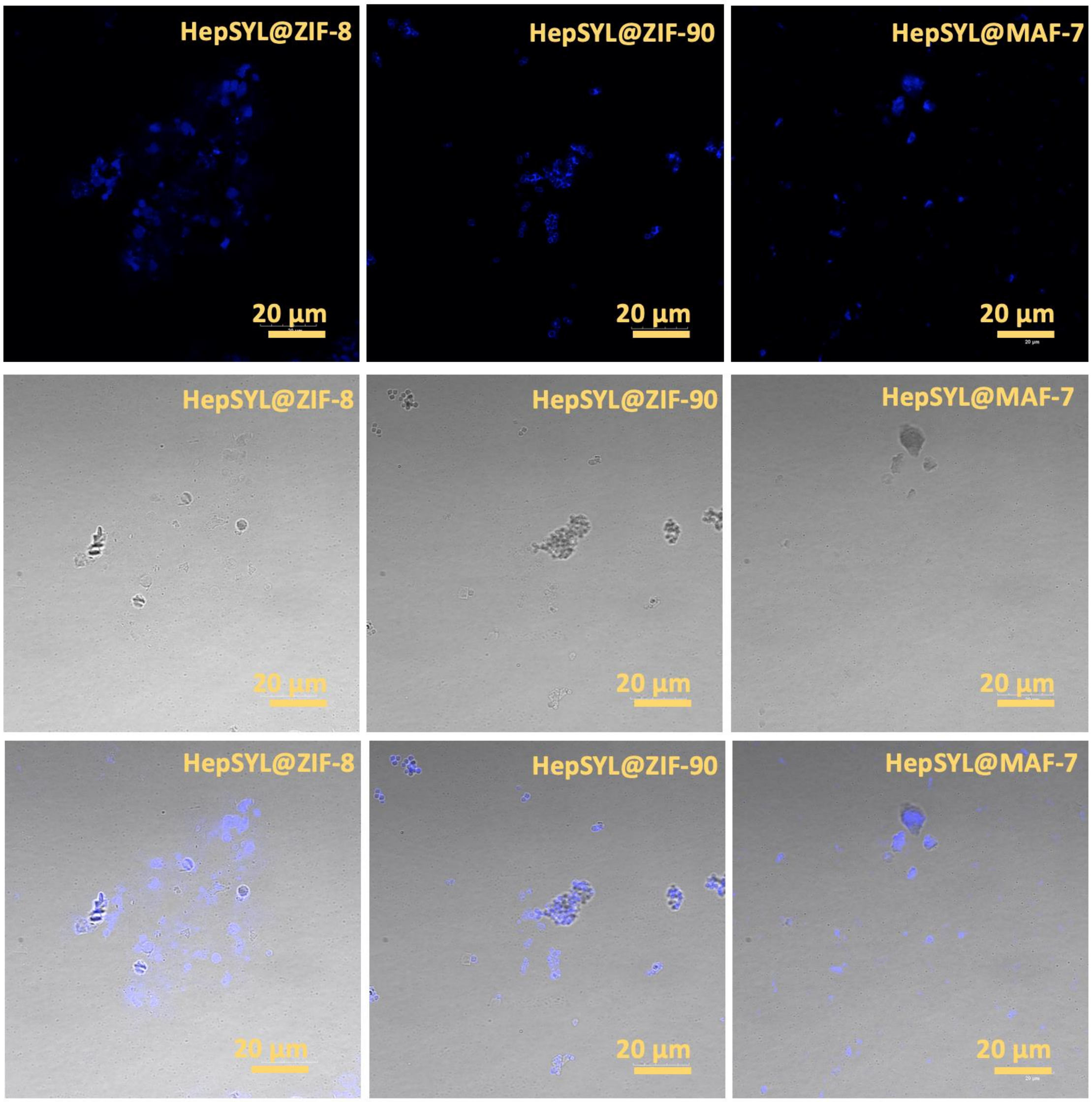

Fig. S21. Confocal laser scanning micrographs taken form the samples HepSYL@ZIF-8, HepSYL@ZIF-90, and HepSYL@MAF-7 showing the fluorescence, bright field and overlay.

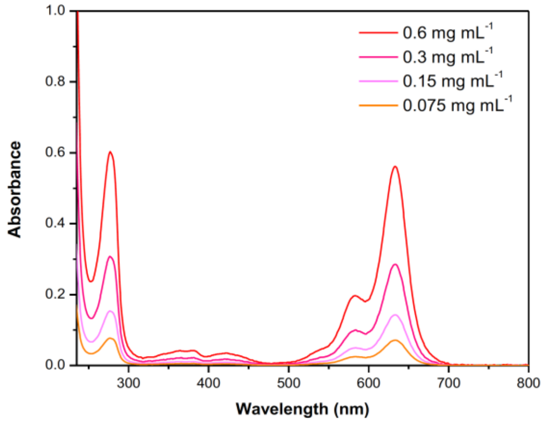

Fig. S22 The UV-Vis absorption spectra of HepSYL.

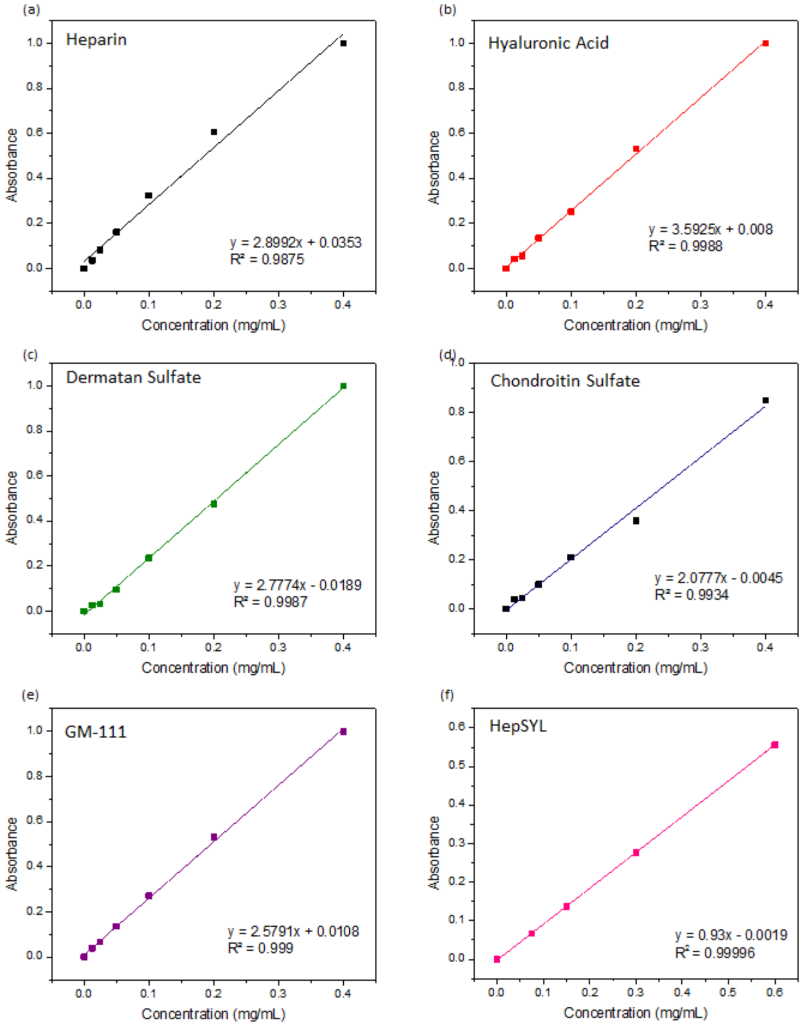

Fig. S23 Calibration curve of GAGs. Calibration curve of Heparin (a) Hyaluronic acid (b) Dermatan sulfate (c) Chondroitin sulfate (d) GM-1111 (e) and HepSYL (f).

Stability of 8DXB3, 90DXB3 and 7DXB3 in SDS and water

To prove the stability of FITC-CMD@ZIF-8 (**8DXB3**), FITC-CMD@ZIF-90 (**90DXB3**), and FITC-CMD@MAF-7 (**7DXB3**) biocomposites in water at pH = 7, the MOF biocomposites were incubated in water for 1h, 5h and 24 h. Then, the supernatant was recovered by centrifugation and analyzed by UV-vis to determine the leaching of cargo (λ_{max} = 490 nm). Additionally, the concentration of Zn^{2+} released upon 24 h of incubation in water at pH = 7 for was determined by ICP-OES.

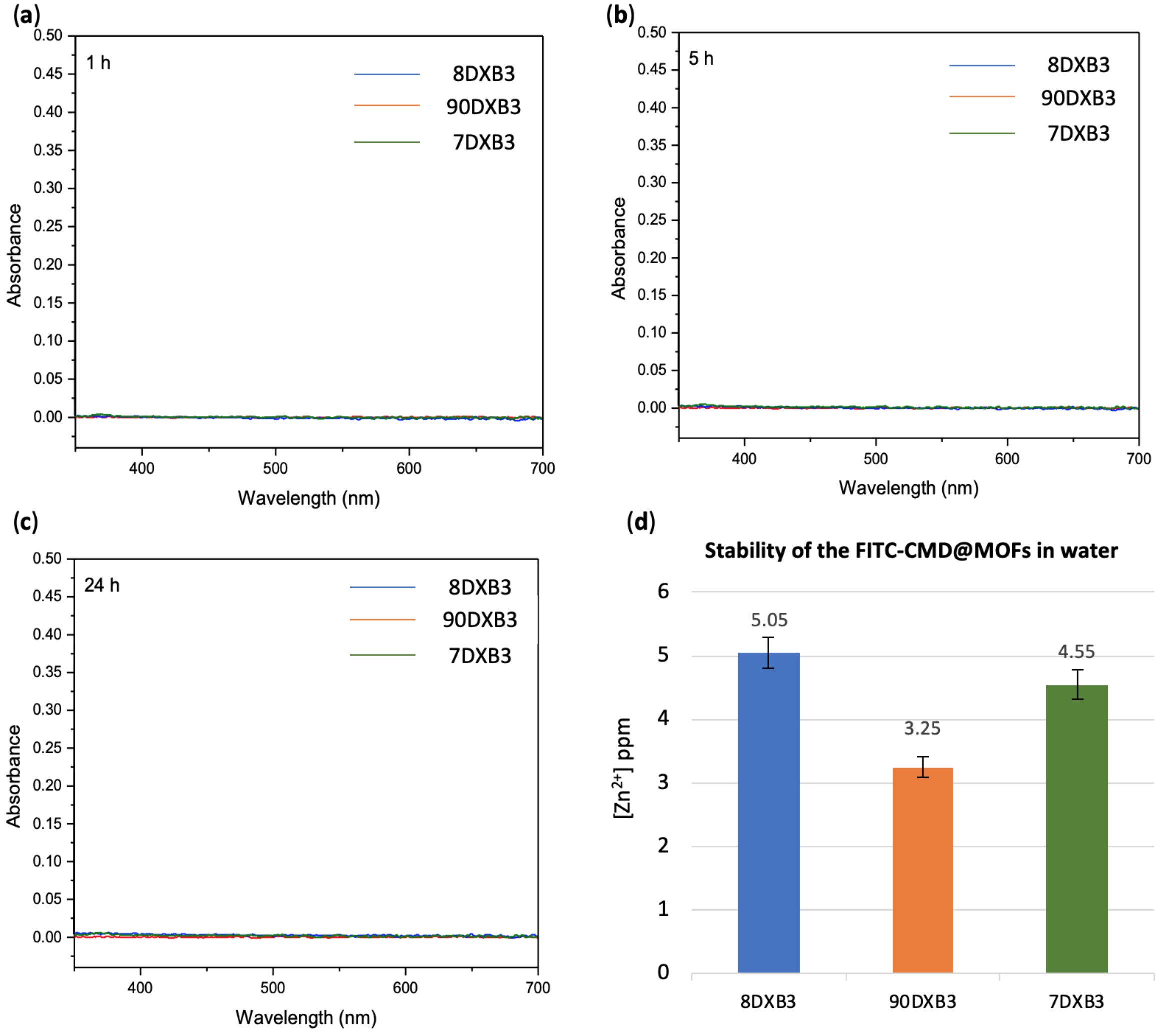

Fig S24. Stability test of FITC-CMD@ZIF-8 (**8DXB3**), FITC-CMD@ZIF-90 (**90DXB3**), and FITC-CMD@MAF-7 (**7DXB3**) biocomposites in water. The FITC-CMD@MOF samples obtained from [FITC-CMD] = 0.36 mg mL^{-1} and Zn^{2+}:L = 1:3.47 were washed with water and then incubated in DI water (2 mL, pH = 7), the supernatant of was analyzed by UV-vis spectroscopy (λ_{max} 490 nm) at different incubation times (a) 1h, (b) 5h, and (c) 24h (d). ICP-OES determination of Zn in the supernatant after the incubation of **8DXB3**, **90DXB3** and **7DXB3** biocomposites in DI water for 24 h that corresponds to a decomposition of 0.37 % (wt) (**8DXB3**), 0.22 % (wt) (**90DXB3**),0.68 % (wt) (**7DXB3**).

To prove the stability of FITC-CMD@ZIF-8 (**8DXB3**), FITC-CMD@ZIF-90 (**90DXB3**), and FITC-CMD@MAF-7 (**7DXB3**) biocomposites in SDS, the MOF biocomposites were incubated in SDS (2.5%, 5%, and 10%), for 30 min. Then, the supernatant was recovered by centrifugation and analyzed by UV-vis to determine the leaching of cargo (λ_{max} = 490 nm). Additionally, the concentration of Zn^{2+} released in the supernatant was determined by ICP-OES.

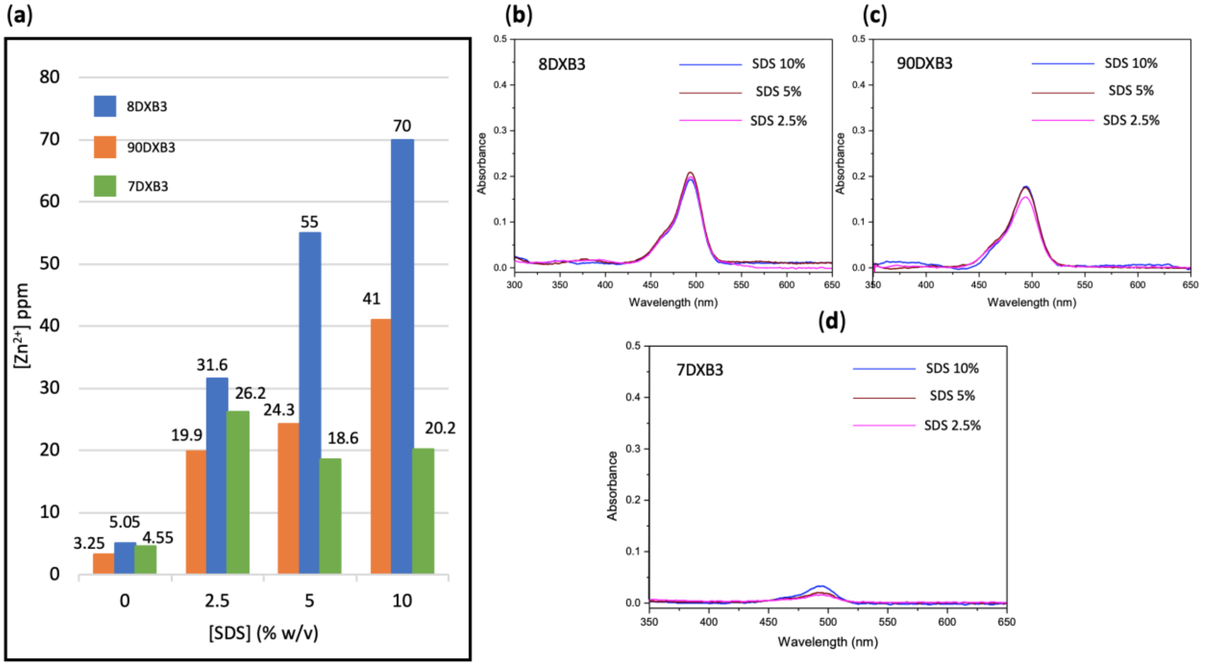

Fig. S25. Stability test of FITC-CMD@MOF biocomposites (**8DXB3**, **90DXB3** and **7DXB3**) in SDS (2.5%, 5%, and 10%). (a) ICP-OES determination of Zn in the supernatant. (b)–(d) UV-vis analysis of the supernatant to prove the release of the cargo FITC.CMD at λ_{max} = 490 nm.

Determination of the anticoagulant activity of HP@MOFs

The anticoagulant activity of heparin was assessed by a chromogenic method for anti-IIa assay using a commercial kit (Iduron ANTI-IIA HEPARIN KIT). This assay is a two-step chromogenic method based on the inhibition of an excess of factor IIa in presence of antithrombin (AT).

Step 1: HP + AT → [AT-HP]

Step 2: [AT-HP] + IIa $_{(excess)}$ → [AT-HP-IIa]$_{(inactive)}$ + IIa $_{(residual)}$

IIa $_{(residual)}$ + IIa substrate $_{(colorless)}$ → pNA $_{(chromophore)}$ ($A_{405\ nm}$) + peptide

The standard solutions were prepared using Heparin sodium salt from porcine intestinal mucosa (>180 USP/mg) purchased from Sigma-Aldrich. (S_0 = 0 IU mL^{-1}, S_1 = 0.1 IU mL^{-1}, S_2 = 0.15 IU mL^{-1}, S_3 = 0.2 IU mL^{-1}, S_4 = 0.25 IU mL^{-1}, S_5 = 0.3 IU mL^{-1}).The HP encapsulated within the MOF shells was released using EDTA solution (40 mM). Then resultant clear solutions were diluted to prepare two test solutions (T_1 = 0.15 IU mL^{-1}, T_2 = 0.30 IU mL^{-1}) for each biocomposite (HP@ZIF-8, HP@ZIF-90, and HP@MAF-7). The dilution factor for each solution was calculated considering: (*i*) the amount of HP encapsulated within each biocomposite (this value was determined by the carbazole assay), (*ii*) the HP activity reported by the manufacturer (> 180 USP mg^{-1}). The standard and test solutions were analyzed following the stablished assay protocol provided by the manufacturer. The

anticoagulant activity of HP@ZIF-8, HP@ZIF-90, and HP@MAF-7 samples was tested by triplicate. The standard curve was obtained plotting the log $A_{\lambda max}$ against [HP] (IU mL^{-1}) the regression line of the standard samples was used to evaluate the relative anticoagulant activity of test samples (Fig. S26a).

Biopreservation experiments

The heparinase I (0.1 IU) was purchased from Iduron, and was supplied as frozen solution. A preliminary experiment was performed in order to determine the time required to complete degradation of the HP used in this work. This kinetic experiment was performed by UV spectroscopy following the formation of uronic acid ($A_{\lambda max}$ = 232 nm) produced during the enzymatic degradation of the HP (Fig. S26c). The experiment was performed at 30 °C in an acetate buffer media (pH = 7, containing sodium acetate 50 mM, and calcium acetate 1mM). The assay was accomplished by mixing 0.8 mL of HP solution (750 μg mL^{-1}) with 0.2 mL of heparinase I solution (50 mu mL^{-1}). The enzymatic kinetic reveals that after 1h of reaction the inactivation of HP proceeds quantitatively. Then, to evaluate the protection capabilities of the MOF materials, each HP@MOFs biocomposite and the free HP were incubated, separately, in acetate buffer media at 30 °C for 1h in presence of heparinase I. Afterwards, the samples were heated at 100 °C for 5 min to inactivate the enzyme. The biocomposite materials were washed with water (2 mL, 3X) and ethanol (2 mL, 3X), and the encapsulated HP was recovered soaking the HP@MOFs biocomposite in EDTA (40 mM) to degrade the MOF matrix. The resultant solutions were diluted to determine the antithrombotic activity of the released HP, using the chromogenic anti-IIa assay (Fig. S26d).

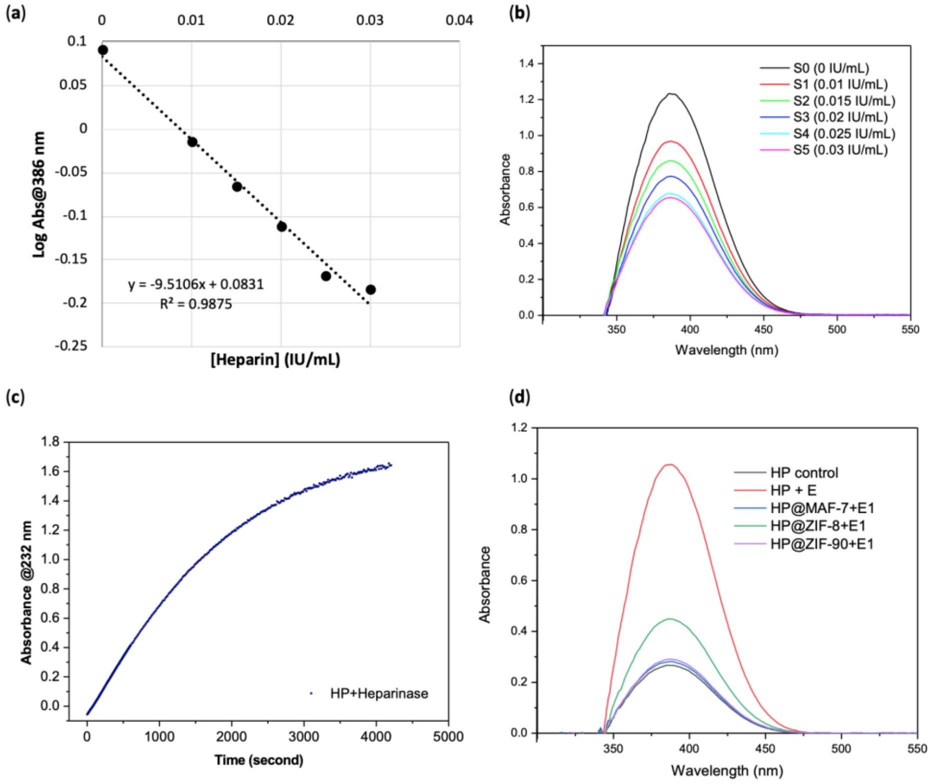

Fig. S26. Chromogenic anti-II assay to determine the anticoagulant activity of HP. (a) calibration curve. (b) Absorbance recorded at 386 nm for standard solutions. (c) Kinetic of the enzymatic degradation of heparin in presence of heparinase I. (d) Absorbance recorded at 386 nm for free HP and encapsulated HP exposed to heparinase I, and the pure HP as a control.

Gas Sorption

Gas adsorption isotherm measurements were performed on an ASAP 2020 Surface Area and Pore Size Analyzer. Samples were activated by heating in vacuum at 100 °C for 3 h under N_2 and 100 °C for 18 h under vacuum ($2X10^{-6}$ mm of Hg). UHP grade (99.999%) N_2 and He were used for all measurements. The temperatures were maintained at 77 K (liquid nitrogen bath).

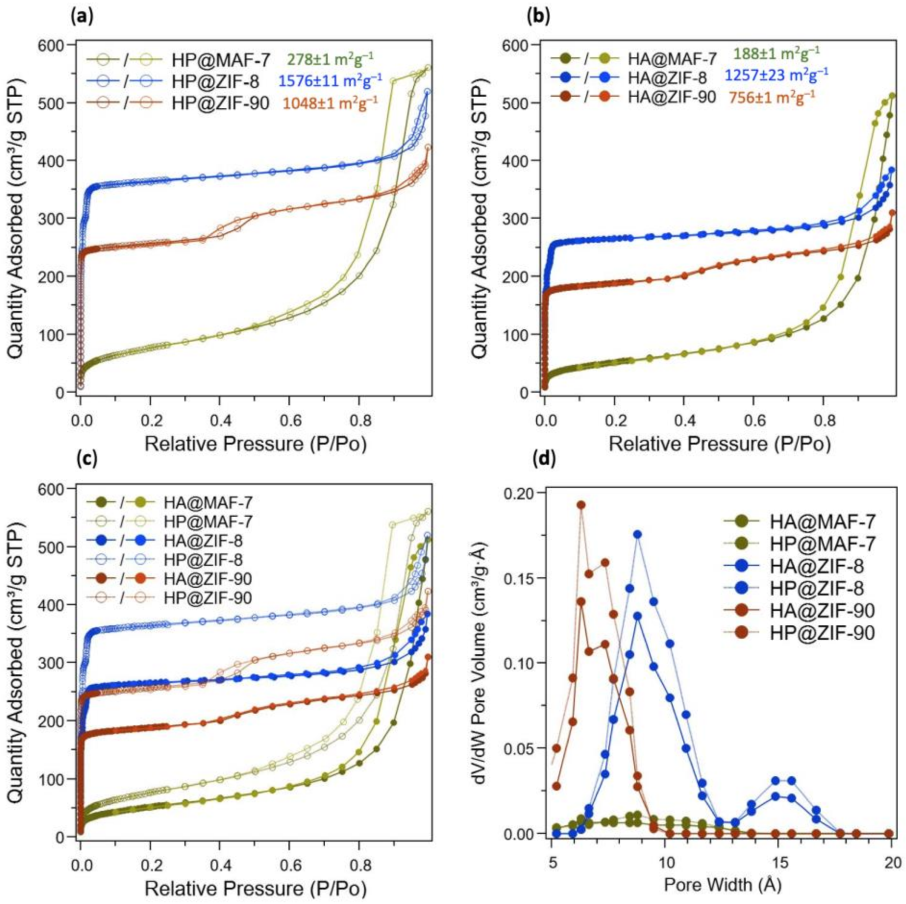

Fig. S27. 77 K N_2 adsorption and desorption isotherms for (a) HP@MOFs and (b) HA@MOFs biocomposites. The calculated BET surfaces areas for samples are listed in the inset. (c) Comparison of the 77 K N_2 adsorption and desorption isotherms obtained varying the biomolecules (HP and HA), and the MOF shell (ZIF-8, ZIF-90 and MAF-7). (d) Calculated pore size distribution curves for HP@MOFs and HA@MOFs biocomposites.

**Sample**	Calculated BET surface area (**m^2g^{-1}**)
HP@ZIF-8	1576±11
HA@ZIF-8	1257±23
**ZIF-8**	**1437±23**
HP@ZIF-90	1048±1
HA@ZIF-90	756±1
**ZIF-90**	**1589±17**
HP@MAF-7	278±1
HA@MAF-7	188±1
**MAF-7**	**1435±23**

Table S3. Comparison of the calculated BET surfaces areas for samples HP@MOFs and HA@MOFs with respect to the neat MOFs (**sod**-ZIF-8, ***sod***-ZIF-90, and ***sod***-MAF-7) prepared under conventional synthetic conditions.

Thermogravimetric analysis (TGA)

TGA data confirmed the estimation made via carbazole assay (Fig. S28). However, the TGA data suggest that GAGs@MOF biocomposites possess a higher amount of zinc cations compared to the pure MOF materials (Fig. S28). This observation may be attributed to the electrostatic interactions between the Zn^{2+} and the negatively charged sulphate and carboxylate groups of the GAGs as reported by Parrish et al.[2] To confirm this hypothesis, we used energy dispersive X-ray spectroscopy (EDS) to determine the elemental composition of the HP@MOF biocomposites and related control samples (i.e. neat MOFs, and free HP) (Fig. S29, ESI†). In all the samples, as shown in plot S29 we observed an excess of Zn associated to the S assigned to sulphate groups. (Fig. S29 and Table S4–S7, ESI†). This excess of Zn validates the hypothesis and explain the TGA results.

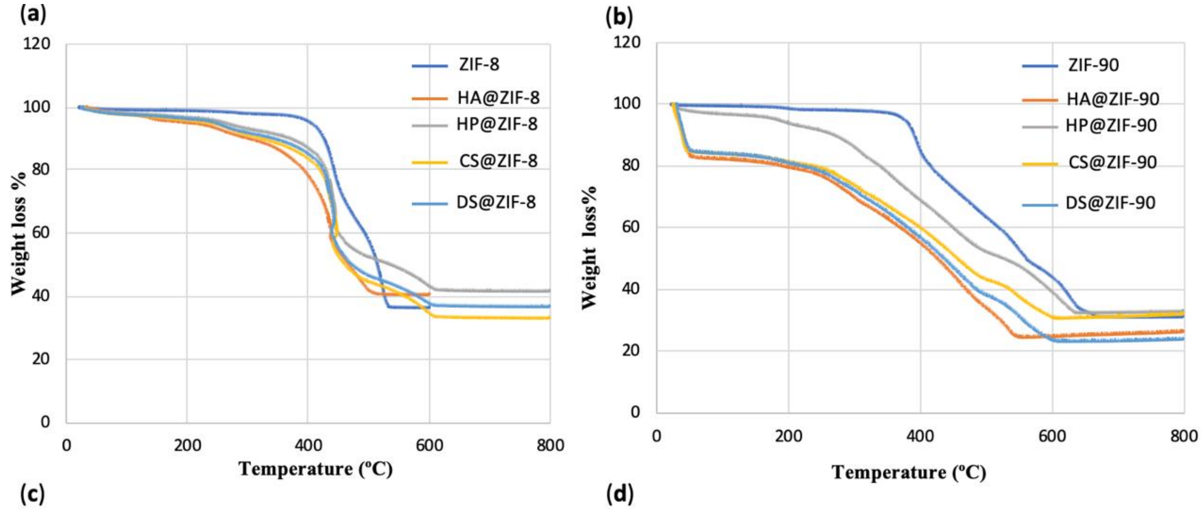

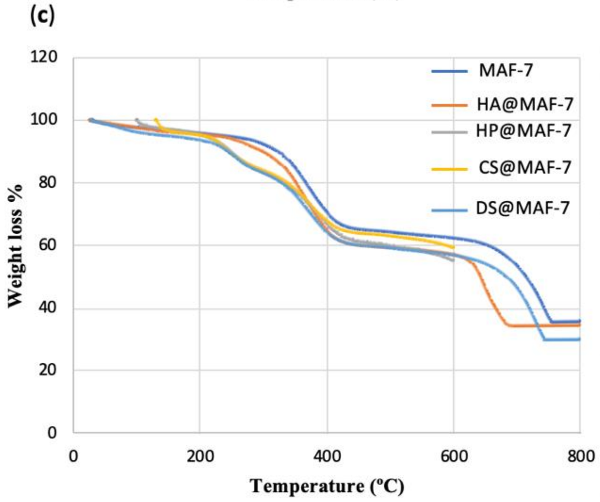

(d)

		% HA	% HP	% CS	% DS	
ZIF-8		7.19	8.14	9.60	6.28	TGA (100-350 ºC approx.)
		7.1 ± 0.5	10.7 ± 0.3	10.4 ± 0.4	6.4 ± 0.7	Carbazole assay
ZIF-90		6.54	5.67	5.58	7.04	TGA (100 – 250 ºC approx.)
		6.1 ± 0.6	6.3 ±0.8	5.3 ± 1.7	8.8 ± 1.0	Carbazole assay
MAF-7		18.08	19.01	19.01	21.1	TGA (100 – 350 ºC approx.)
		24.1 ± 5.3	21.5 ± 1.2	20.1 ± 1.3	21.4 ±0.7	Carbazole assay

Fig. S28. Thermogravimetric analysis (TGA) of showing the thermal decomposition of (a) neat ZIF-8 and GAG@ZIF-8 biocomposites, (b) neat ZIF-90 and GAG@ZIF-90 biocomposites, (c) neat MAF-7 and GAG@MAF-7 biocomposites. (d) Estimation loading capacity using two different techniques: TGA and carbazole assay (data given in weight % referenced to the 100% of the biocomposite).

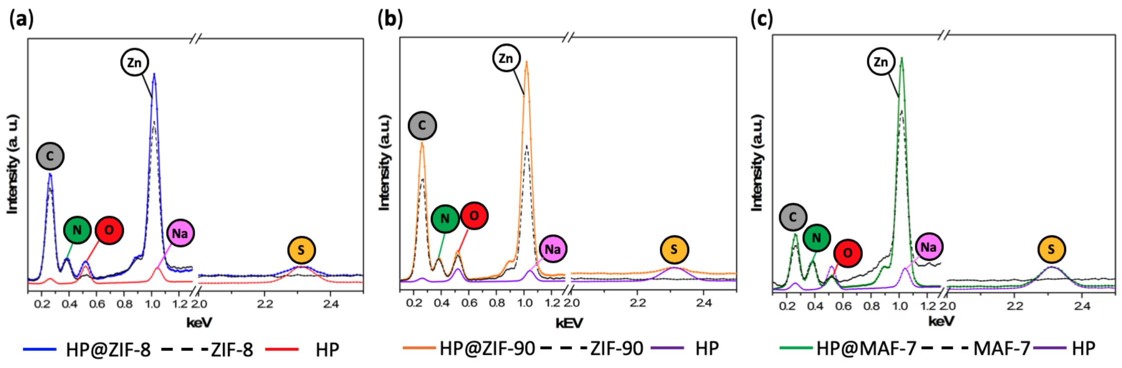

Fig. S29. EDS elemental composition of HP@MOF biocomposites and their corresponding neat MOFs. EDS spectrum of (a) HP@ZIF-8, (b) HP@ZIF-90, and (c) HP@MAF-7 and the elemental contributions of their corresponding constituents pure HP and ZIF-8, ZIF-90 and MAF-7; respectively.

Average(wt%)							
Element (wt%)	HP	HP@MAF-7	MAF-7	HP@ZIF-8	ZIF8	HP@ZIF-90	ZIF-90
C	29.64	30.39	30.01	38.87	40.76	41.73	42.210
O	47.07	10.30	5.24	11.49	3.65	13.20	17.400
Na	11.80	0.00	0.00	0.00	0.00	0.00	0.000
S	11.49	1.98	0.00	0.79	0.00	0.38	0.000
Zn	0.00	19.18	2.72	21.18	21.78	21.72	14.15
N	0.00	36.39	34.18	27.65	33.81	22.96	26.24
Cl	0.00	1.78	2.72	0	0	0	0

Table S4. Elemental composition of HP@MOFs biocomposites and their corresponding neat MOFs obtained from EDS analysis

Contribution to the elemental composition of HP@ZIF-8					
Element	(wt%) from ZIF-8	(wt%) from HP	solvent and other impurities	Excess of Zn^{2+}(wt%)	HP@ZIF-8 total
C	33.33	2.05	3.49		38.87
O		3.25	8.24		11.49
S		0.79	0.00		0.79
Zn	17.81			3.37	21.18
N	27.65				27.65
TOTAL	78.80	6.09	11.74	3.37	100.00

Table S5. Estimation of the excess of Zn^{2+} in HP@ZIF-8, based on the elemental contribution of neat ZIF-8 and pure HP.

Contribution to the elemental composition of HP@ZIF-90					
Element	(wt%) from ZIF-90	(wt%) from HP	solvent and other impurities	Excess of Zn^{2+}(wt%)	HP@ZIF-90 total
C	40.75	0.98	3.49		41.73
O	11.65	1.56	8.24		13.21
S		**0.38**	0.00		0.38
Zn	12.38			9.34	21.72
N	22.96				22.96
TOTAL	87.74	2.92	11.74	9.34	100.00

Table S6. Estimation of the excess of Zn^{2+} in HP@ZIF-90, based on the elemental contribution of neat ZIF-90 and pure HP.

Contribution to the elemental composition of HP@MAF-7					
Element	(wt%) from MAF-7	(wt%) from HP	solvent and other impurities	Excess of Zn^{2+}(wt%)	HP@MAF-7 total
C	25.28	5.11	0.00		30.39
O		8.11	2.18		10.30
S		**1.98**	0.00		1.98
Zn	2.89			16.28	19.18
N	36.39				36.39
Cl			1.78		1.78
TOTAL	64.55	15.20	3.96	16.28	100.00

Table S7. Estimation of the excess of Zn^{2+} in HP@MAF-7, based on the elemental contribution of neat MAF-7 and pure HP.

Estimation of the USP units of heparin released form the maximum safe dosage of HP@MOF

Herein, we provide an estimation of the USP units of heparin released form the maximum safe dosage of HP@MOF biocomposites. Based on the cytotoxicity of ZIF-8 (30 mg L^{-1}),[3] we can estimate the threshold concentration for ZIF-90 (32 mg L^{-1}), and MAF-7 (30 mg L^{-1}) to ensure the biocompatibility of those materials (IC20) (see Table S8 and Fig S30). Considering the amount of HP encapsulated in 100 mg of biocomposite, we calculated the USP units of HP released from the maximum dose of HP@MOF (Table S8, Fig. S30). According to the current dose regulations for heparin,[4] MAF-7 seems to be a suitable carrier for intravenous injection of HP. The particle size of this biocomposite is compatible with this administration route.[5]

Sample	%HP Loading Capacity	Maximum dosage of HP@MOF (mg L^{-1})	Maximum dosage of HP@MOF (mg)[a]	USP units of heparin encapsulated in the maximum dosage[b]
HP@ZIF-8	8.14	32.7	163.3	2392.5
HP@ZIF-90	5.67	33.9	169.6	1731.1
HP@MAF-7	19.01	37.0	185.2	6337.4

Table S8. Estimation of USP units of Heparin released from the maximum safe dosage of each HP@MOF biocomposites, [a] Average body weight 70 kg equivalent to blood volume of 5 L, [b] The HP used in this work has 180 USP mg^{-1}.

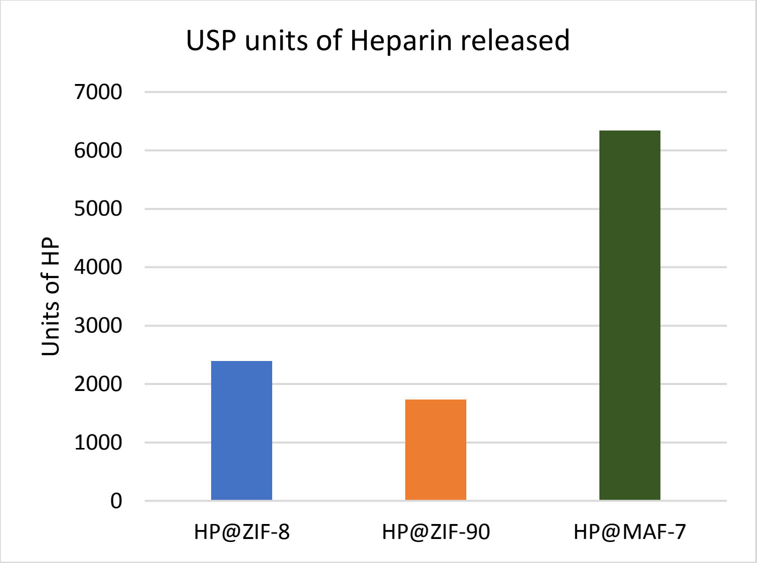

Fig. S30. Estimation of USP units of HP released from HP@MOFs

Type of system	Material	Type of immobilization	EE(%)	Loading capacity (wt%)	Protection properties	Ref
MOFs	This work ZIF-8	Encapsulation	100	8.14	Protection of heparin from Heparinase I enzyme	
	This work ZIF-90	Encapsulation	60	5.67	Protection of heparin from Heparinase I enzyme	
	This work MAF-7	Encapsulation	98	19	Protection of heparin from Heparinase I enzyme	
	MIL-101(Fe)	Adsorption	Not stated	15	Not stated	6
Silica	Mesoporous Silica nanoparticle (MSN)	Encapsulation	Not stated	Not stated	Not stated	7
	Silica Xerogel	Sol-gel	Not stated	13.6	Not stated	8
	ammoniated-hollow mesoporous silica (A-HMS)	Adsorption	Not stated	9	Not stated	9
	ammoniated-magnetic mesoporous silica (A-MMS)	Adsorption	Not stated	1.5	Not stated	9
	(PEDOT/MS/MnO_2)	Adsorption	Not stated	5.6	Not stated	10
	PEDOT/MS	Adsorption	Not stated	5.8	Not stated	10
	PEDOT/MNO_2	Adsorption	Not stated	0.6	Not stated	10
Polymer	Eudragit RS (RS)	Encapsulation	59	6.57	Not stated	11
	PLGA	Encapsulation	14	1.6	Not stated	11
	Poly caprolactone	Encapsulation	8	0.9	Not stated	11
	Eudragit RL (RL)	Encapsulation	97	10.8	Not stated	11
	RL/PCL	Encapsulation	53	5.9	Not stated	11
	RS/RL/PLGA	Encapsulation	38	4.2	Not stated	11
	RS/PLGA	Encapsulation	36	3.9	Not stated	11
	N-trimethyl chitosan (TCM)	Ionotropic gelation	71.9	Not stated	Protection of heparin from gastrointestinal tract pH gastric fluid (SGF, pH 1.2) and simulated intestinal fluid (SIF, pH 7.4)	12
	Chitosan (CS)	Ionotropic gelation	72.6	Not stated	Protection of heparin from SGF, pH (1.2) and SIF (pH 7.4) (lower than TCM)	12
	PMMA-b-PMAETMA	Encapsulation	98	16.4	Not stated	13
	PHB-HV/PEG	Encapsulation	59	Not stated	Not stated	14
	PCL	Encapsulation	Not stated	0.5	Not stated	15
	PCL-α-TCP	Encapsulation	Not stated	2	Not stated	16
	PLGA	Encapsulation	14.9	Not stated	Not stated	17
	RS	Encapsulation	98.1	Not stated	Not stated	18
	RS/PLGA	Encapsulation	96.9	Not stated	Not stated	18
	PLGA	Encapsulation	38.6	Not stated	Not stated	18
	PLLA-PEG-PLLA	Encapsulation	15.8	0.52	Not stated	19
	EDC/NHS-crosslinked collagen	Grafting	Not stated	5.5	Not stated	20
	Thermosensitive hydrogel (TSH)	Absorption	Not stated	15.3	Not stated	21
	Thiolated Chitosan (TCS)	Grafting	97.91	15.14	Protection of heparin from stomach acid and digestive enzyme degradation (pepsin)	22
	PLGA:E-RLPO	Encapsulation	92.1	Not stated	Not stated	23
	RL	Encapsulation	80	8.87	Not stated	24
	RS/gelatin A	Encapsulation	67	7.4	Not stated	24
	PLGA/Gelatin A	Encapsulation	58	6.5	Not stated	24
	PCL/Gelatin A	Encapsulation	58	6.4	Not stated	24
others	Liposomes	Encapsulation	48.3	Not stated	Protection of heparin from Temperature (40 °C)*	25
	Thrombin-responsive polymer	Grafting	Not stated	Not stated	Not stated	26
	Erythrocytes	Encapsulation	44	Not stated	Not stated	27

Table S9. Comparative overview of the properties reported for other drug delivery systems designed for heparin release. MS= mesoporous silica, CL=cargo loading, PEDOT = poly(3,4-ethylenedioxythiophene), PLGA = poly(lactic-co-glycolic acid), PMMA-b-PMAETMA = poly(methyl methacrylate-b-trimethyl aminoethyl methacrylate), PHB = polyhydroxybutyrate-co-hydroxyvalerate, PEG = polyethylene glycol, PCL = poly(e-caprolactone), TCP = tricalcium phosphate, PLLA = poly(L-lactic acid). When needed, we used the efficacy of our heparin (180 USP/mg) for the calculation of the lading capacity.

*from Aldrich, our Heparin can be treated at 120 °C

References

1 E. Astria, M. Thonhofer, R. Ricco, W. Liang, A. Chemelli, A. Tarzia, K. Alt, C. E. Hagemeyer, J. Rattenberger, H. Schroettner, T. Wrodnigg, H. Amenitsch, D. M. Huang, C. J. Doonan and P. Falcaro, *Materials Horizons*, 2019, **6**, 969–977.
2 R. F. Parrish and W. R. Fair, *Biochem. J.*, 1981, **193**, 407–410.
3 M. Hoop, C. F. Walde, R. Riccò, F. Mushtaq, A. Terzopoulou, X.-Z. Chen, A. J. deMello, C. J. Doonan, P. Falcaro, B. J. Nelson, J. Puigmartí-Luis and S. Pané, *Applied Materials Today*, 2018, **11**, 13–21.
4 M. A. Smythe, J. Priziola, P. P. Dobesh, D. Wirth, A. Cuker and A. K. Wittkowsky, *J Thromb Thrombolysis*, 2016, **41**, 165–186.
5 T. Simon-Yarza, A. Mielcarek, P. Couvreur and C. Serre, *Adv. Mater.*, 2018, **30**, 1707365.
6 V. V. Vinogradov, A. S. Drozdov, L. R. Mingabudinova, E. M. Shabanova, N. O. Kolchina, E. I. Anastasova, A. A. Markova, A. A. Shtil, V. A. Milichko, G. L. Starova, R. L. M. Precker, A. V. Vinogradov, E. Hey-Hawkins and E. A. Pidko, *J. Mater. Chem. B*, 2018, **6**, 2450–2459.
7 F. Wu, T. Xu, G. Zhao, S. Meng, M. Wan, B. Chi, C. Mao and J. Shen, *Langmuir*, 2017, **33**, 5245–5252.
8 M. S. Ahola, E. S. Säilynoja, M. H. Raitavuo, M. M. Vaahtio, J. I. Salonen and A. U. O. Yli-Urpo, *Biomaterials*, 2001, **22**, 2163–2170.
9 S. Hu, S. Shao, H. Chen, J. Sun, J. Zhai, H. Zheng, M. Wan, Y. Liu, C. Mao and J. Zhao, *J. Phys. Chem. C*, 2018, **122**, 9680–9687.
10 Q. Wang, Y. Wang, B. Guo, S. Shao, Y. Yu, X. Zhu, M. Wan, B. Zhao, C. Bo and C. Mao, *J. Mater. Chem. B*, 2019, **7**, 2688–2695.
11 Y. Jiao, N. Ubrich, M. Marchand-Arvier, C. Vigneron, M. Hoffman, T. Lecompte and P. Maincent, *Circulation*, 2002, **105**, 230–235.
12 R. Paliwal, S. R. Paliwal, G. P. Agrawal and S. P. Vyas, *International Journal of Pharmaceutics*, 2012, **422**, 179–184.
13 F. Reyes-Ortega, G. Rodríguez, M. R. Aguilar, M. Lord, J. Whitelock, M. H. Stenzel and J. San Román, *J. Mater. Chem. B*, 2013, **1**, 850–860.
14 A. Monnier, C. Rombouts, D. Kouider, I. About, H. Fessi and N. Sheibat-Othman, *International Journal of Pharmaceutics*, 2016, **513**, 49–61.
15 E. Luong-Van, L. Grøndahl, K. N. Chua, K. W. Leong, V. Nurcombe and S. M. Cool, *Biomaterials*, 2006, **27**, 2042–2050.
16 M. Alehosseini, N. Golafshan, M. Kharaziha, M. Fathi and H. Edris, *Macromol. Biosci.*, 2018, **18**, 1800020.
17 Lee, Byong Taek, *Bulletin of the Korean Chemical Society*, 2011, **32**, 1465–1470.
18 A. Lamprecht, P. Koenig, N. Ubrich, P. Maincent and D. Neumann, *Nanotechnology*, 2006, **17**, 3673–3680.
19 X. Luo, D. Qiu, B. He, L. Wang and J. Luo, *Macromol. Biosci.*, 2006, **6**, 373–381.
20 M. J. B. Wissink, R. Beernink, J. S. Pieper, A. A. Poot, G. H. M. Engbers, T. Beugeling, W. G. van Aken and J. Feijen, *Biomaterials*, 2001, **22**, 151–163.
21 A. Gutowska, Y. H. Bae, J. Feijen and S. W. Kim, *Journal of Controlled Release*, 1992, **22**, 95–104.
22 B. Fan, Y. Xing, Y. Zheng, C. Sun and G. Liang, *Drug Delivery*, 2016, **23**, 238–247.

23 V. Ganti, A. Mengesha, J. Marszalek and B.-B. Youan, *Acta Pharmaceutica*, 2010, **60**, 281–293.
24 Y. Jiao, N. Ubrich, V. Hoffart, M. Marchand-Arvier, C. Vigneron, M. Hoffman and P. Maincent, *Journal of Pharmaceutical Sciences*, 2002, **91**, 760–768.
25 K. Vaghasiya, A. Sharma, K. Kumar, E. Ray, S. Adlakha, O. P. Katare, S. K. Hota and R. K. Verma, *ACS Biomater. Sci. Eng.*, 2019, **5**, 6617–6631.
26 Y. Zhang, J. Yu, J. Wang, N. J. Hanne, Z. Cui, C. Qian, C. Wang, H. Xin, J. H. Cole, C. M. Gallippi, Y. Zhu and Z. Gu, *Adv. Mater.*, 2017, **29**, 1604043.
27 H. G. Eichler, W. Schneider, G. Raberger, S. Bacher and I. Pabinger, *Res. Exp. Med.*, 1986, **186**, 407–412.
